# Ambrisentan attenuates cisplatin-related mitochondrial dysfunction in the heart via regulation of p53 and NF-κB signaling

**DOI:** 10.1038/s41598-026-44822-9

**Published:** 2026-03-24

**Authors:** Hnin Ei Ei Khine, Supachoke Mangmool, Warisara Parichatikanond

**Affiliations:** 1 Department of Pharmacology, Faculty of Pharmacy, Mahidol University, Bangkok, 10400 Thailand; 2 Department of Pharmaceutical Care, Faculty of Pharmacy, Chiang Mai University, Chiang Mai, 50200 Thailand; 3 Centre of Biopharmaceutical Science for Healthy Ageing, Faculty of Pharmacy, Mahidol University, Bangkok, 10400 Thailand; 4 Centre of Molecular Targeting and Integrated Drug Development, Faculty of Pharmacy, Mahidol University, Bangkok, 10400 Thailand

**Keywords:** Ambrisentan, Cisplatin, ET_A_ receptor antagonist, Mitochondrial dysfunction, NF-κB, p53, Biochemistry, Cancer, Cardiology, Cell biology, Drug discovery, Molecular biology

## Abstract

Ambrisentan, an endothelin receptor A (ET_A_) antagonist, confers beneficial effects in cardiovascular diseases, but its role in mitigating chemotherapy-induced cardiotoxicity remains unexplored. This study investigates the protective effects of ambrisentan against cisplatin-induced cardiotoxicity in H9c2 cardiomyoblasts, focusing on its regulatory role via the p53 and NF-κB signaling pathways. Ambrisentan reduced cisplatin-induced apoptosis by downregulating caspase-3/7 activity and BAX expression while upregulating Bcl-2. Cisplatin-induced inflammation was also attenuated by ambrisentan through the reduction of TNFα and IL6 levels. Furthermore, ambrisentan protected cells against cisplatin-induced mitochondrial damage by lowering ROS levels, promoting mitochondrial fusion (OPA1, MFN1), inhibiting fission (DNM1, FIS1), and enhancing mitochondrial biogenesis (ATP5A, NRF1, PGC1α). Structured illumination microscopy revealed that ambrisentan preserved tubular mitochondrial morphology and reversed cisplatin-induced fragmentation. In addition, ambrisentan restored mitochondrial respiration and glycolysis, evidenced by improved oxygen consumption rate and extracellular acidification rate, along with increased pro-survival molecules (p-Akt and p-Erk1/2), under cisplatin-mediated stress. Mechanistically, pharmacological inhibition of p53 and NF-κB enhanced the protective effects of ambrisentan, whereas their activation exacerbated cisplatin-induced cardiac injury. Therefore, ambrisentan may serve as a potential agent for suppressing apoptosis, inflammation, and mitochondrial impairments, offering protection against cisplatin-associated cardiotoxicity through modulation of the p53 and NF-κB pathways.

**Supplementary Information:**

The online version contains supplementary material available at 10.1038/s41598-026-44822-9.

## Introduction

Heart failure is a complex clinical syndrome that can develop either as a primary condition or as a secondary complication resulting from various underlying diseases, such as hypertension, diabetes, myocardial infarction, or even as a consequence of certain drug therapies, including chemotherapy^1^. Mitochondria, known as the powerhouse of eukaryotic cells, are essential for cellular energy production, particularly in high-energy-demand organs like the heart^2^. In cardiomyocytes, mitochondria generate adenosine triphosphate (ATP) primarily through oxidative phosphorylation, which supports key physiological processes such as muscle contraction, ion pumping, and cellular signaling^3^. Mitochondrial dysfunction in the heart is characterized by reduced ATP production, altered mitochondrial morphology, activation of apoptotic pathways, oxidative stress, and calcium dysregulation, all of which contribute to the development of heart failure by decreasing myocardial contractility, increasing the risk of arrhythmias, and worsening overall cardiac function^4–6^. Understanding the role of mitochondria is important for protecting cardiac function, especially in the context of chemotherapy, where certain drugs, such as cisplatin, can damage mitochondria and further exacerbate cardiac dysfunction^7–9^.

Cisplatin, a widely used chemotherapeutic agent for treating solid tumors, is particularly known for inducing mitochondrial dysfunction in cardiac cells. Upon entering cardiac cells, cisplatin disrupts mitochondrial function, causing membrane depolarization and impaired respiration^10–12^, which in turn activates cellular responses, including apoptosis and inflammation, primarily mediated by the tumor protein p53 (p53) and nuclear factor-kappa B (NF-κB) signaling pathways, ultimately contributing to cardiotoxicity^10,13,14^. The p53 pathway responds to cellular stress by promoting apoptosis through the downregulation of anti-apoptotic B-cell lymphoma 2 (Bcl-2) and the upregulation of pro-apoptotic Bcl-2-associated X protein (BAX), leading to mitochondrial membrane permeabilization and caspase activation^15,16^. In parallel, cisplatin activates the NF-κB pathway, triggering the release of inflammatory cytokines, including tumor necrosis factor-alpha (TNFα) and interleukin-6 (IL6), which further exacerbate myocardial injury. These consequences create a vicious cycle of mitochondrial dysfunction, apoptosis, and inflammation, worsening cardiac function and increasing the risk of heart failure^17,18^. Altogether, these interconnected pathways highlight the multifactorial nature of cardiac injury mediated by cisplatin, suggesting that targeting the p53 and NF-κB signaling pathways could offer therapeutic avenues to reduce the cardiotoxic effects of cisplatin while improving its safety and clinical efficacy^10,13^.

Endothelin (ET) receptors are G protein-coupled receptors that mediate the actions of endothelin-1 (ET-1), a potent vasoconstrictor peptide known to induce oxidative stress, mitochondrial damage, and deterioration of cardiac function^19,20^. The two subtypes of ET receptors are ET_A_ and ET_B_ receptors, and both are expressed in cardiac cells such as cardiomyocytes and smooth muscle cells, where they regulate vasoconstriction and cardiac remodeling, ensuring proper cardiac function^21,22^. Several lines of evidence have reported that ET receptor antagonists (ERAs) reduce mitochondrial oxidative stress, support mitochondrial bioenergetics, and enhance the dynamics of mitochondrial fusion and fission, which are critical for maintaining mitochondrial quality control and function^23–26^. ERAs regulate mitochondrial dynamics by enhancing fusion through mitofusin 1 (MFN1) and optic atrophy 1 (OPA1), reducing fission via fission protein 1 (FIS1) and dynamin-related protein 1 (DNM1), and promoting biogenesis by upregulating peroxisome proliferator-activated receptor gamma coactivator-1 alpha (PGC1α) and nuclear respiratory factor 1 (NRF1). Consequently, these actions improve mitochondrial function and contribute to cardioprotection and nephroprotection by maintaining mitochondrial integrity^27–29^.

Ambrisentan is a member of the ERA drug class that selectively targets the vasoconstrictor ET_A_ receptor and is primarily indicated for treating pulmonary arterial hypertension by improving vascular tone and lowering pulmonary vascular resistance^30^. Ambrisentan also reduced ET-1-induced cellular damage, fibroblast activation, and myofibroblast differentiation, while preserving mitochondrial integrity and bioenergetics through the Erk signaling pathway^31^. Moreover, ambrisentan has been shown to counteract oxidative stress, mitochondrial abnormalities, and mitochondrial membrane potential loss in cardiomyocytes under hyperglycemic conditions^32^. A recent study demonstrated that ambrisentan protects against oxidative stress and apoptosis through the p53 axis, especially in models of acute kidney injury in response to cisplatin^33^. However, the potential protective effects of ambrisentan and its underlying signaling pathways in cisplatin-induced cardiac damage have not been identified so far. Accordingly, this study investigated the cardioprotective effects of ambrisentan against mitochondrial dysfunction, inflammation, and apoptosis in cisplatin-induced cardiotoxicity, with particular emphasis on the involvement of p53 and NF-κB signaling pathways in H9c2 cardiomyoblasts.

## Materials and methods

### Materials

Ambrisentan, pifithrin-alpha hydrobromide (PIFα, a p53 inhibitor), nutlin-3 (NUT3, a p53 activator), JSH-23 (JSH, a NF-κB inhibitor), and NF-κB activator 1 (NA1, a NF-κB activator) were purchased from MedChemExpress (Monmouth Junction, NJ, USA). Cisplatin was obtained from Thermo Fisher Scientific (Waltham, MA, USA). Skim milk powder and 3-(4,5-dimethylthiazol-2yl)-2,5-diphenyltetrazolium bromide (MTT) were ordered from Sigma-Aldrich (Saint Louis, MO, USA). Dulbecco’s Modified Eagle Medium (DMEM), 0.25% trypsin–EDTA solution, penicillin/streptomycin/amphotericin B (P/S/A), fetal bovine serum (FBS), and other cell culture reagents were acquired from Gibco (Grand Island, NY, USA).

### Cell culture

H9c2 rat cardiomyoblast cells (catalog number CRL-1466), obtained from the American Type Culture Collection (ATCC, Manassas, VA, USA), are immortalized cells exhibiting cardiac characteristics that are extensively used to study mitochondrial function in models of cardiac injury^34^; in this study, they were used between passages 10 and 20. Cells were maintained in DMEM medium supplemented with 10% FBS and 1% P/S/A and incubated at 37 °C in a 5% CO_2_ atmosphere. Upon reaching 80% confluence, cells were passaged using 0.25% trypsin–EDTA to sustain their exponential growth phase.

### Cell viability

Cell viability was assessed using the MTT assay^35^. Cells were seeded at a density of 1 × 10^4^ cells/well in 96-well plates overnight. Cells were pretreated with PIFα (20 µM), NUT3 (5 µM), JSH (10 µM), or NA1 (1 µM) for 1 h, followed by incubation with ambrisentan (1 µM) for 3 h and subsequently exposed to cisplatin (10 µM) for 24 h. Afterward, the medium was replaced with MTT solution (0.5 mg/mL) and incubated in the dark for 3 h. The formazan product was solubilized in dimethyl sulfoxide and absorbance was measured at 570 nm using a Synergy HTX microplate reader (BioTek, Winooski, VT, USA). The results were calculated as a percentage of absorbance in treated groups compared with vehicle-treated controls.

### Caspase-3/7 activity

The apoptotic level in H9c2 cells was further confirmed using the Caspase-Glo 3/7 assay kit (Promega, Madison, WI, USA). Cells were seeded at a density of 1 × 10^4^ cells/well in 96-well plates overnight. After the specified treatments, 100 µL of Caspase-Glo 3/7 luminescent reagent was added to each well and incubated for 30 min in the dark with gentle shaking. Luminescence was then measured by a Synergy HTX microplate reader (λ_Em_: 528 nm). The results were calculated as a percentage of luminescence in treated groups compared with vehicle-treated controls.

### Secreted TNFα levels by ELISA

The intracellular inflammatory cytokine TNFα levels were determined using a rat TNFα ELISA kit (ELK Biotechnology, Denver, CO, USA). Cells were seeded at a density of 2 × 10^5^ cells/well in 6-well plates overnight. After treatments, the culture medium was collected. Then, 100 μL of each culture medium sample and each standard were added into the appropriate wells according to the manufacturer’s instructions. Once the reaction was complete, absorbance was measured at 450 nm using a Synergy HTX microplate reader. The TNFα concentrations in the culture medium were then calculated based on the standard curve.

### Mitochondrial and intracellular ROS levels

The mitochondrial reactive oxygen species (ROS) generation was evaluated using MitoSOX Red (Invitrogen, Waltham, MA, USA)^36^. Cells were seeded at a density of 1 × 10^5^ cells/well on 2% gelatin-coated coverslips in 12-well plates overnight. After the indicated treatments, cells were washed with phosphate-buffered saline (PBS) and incubated with 2.5 μM MitoSOX at 37 °C for 15 min in the dark. Following another wash, the coverslips with stained cells were mounted with Prolong Diamond Antifade Mountant containing 4,6-diamidino-2-phenylindole (DAPI) (Invitrogen). Fluorescent signals were captured using a Nikon Eclipse Ti2E fluorescence inverted microscope (Nikon Instruments, Melville, NY, USA) (20 × magnification; λ_Ex_/λ_Em_: 396/610 nm). Fluorescence intensity was measured on a per-cell basis using ImageJ software (Java 1.8.0_172, National Institutes of Health, Bethesda, MD, USA), analyzing at least 100 cells per experimental group across multiple randomly selected image frames.

The intracellular ROS production was quantified using 2′,7′-dichlorodihydrofluorescein diacetate (DCFH-DA) (Sigma-Aldrich)^36^. Cells were seeded at a density of 2 × 10^4^ cells/well in 96-well plates overnight. After the designated treatments, cells were washed with PBS and incubated with DCFH-DA (10 μM) at 37 °C for 30 min in the dark. The fluorescence intensity of DCF was quantified by a Synergy HTX microplate reader (λ_Ex_/λ_Em_: 485/528 nm). The results were calculated as a percentage of fluorescence intensity in treated groups compared with vehicle-treated controls.

### Mitochondrial morphology by structured illumination microscopy

To evaluate mitochondrial architecture, cells were stained with MitoTracker Red CMXRos (Invitrogen), a fluorogenic dye that specifically targets mitochondria^32^. Cells were seeded onto gelatin-coated coverslips at a density of 1 × 10^5^ cells/well in 12-well plates and incubated overnight. Following treatment, cells were rinsed with PBS and incubated with 50 nmol/L MitoTracker Red CMXRos for 30 min at 37 °C in the dark. Fluorescent images were acquired using a structured illumination microscope (SIM), ZEISS Lattice SIM 5 (Carl Zeiss Microscopy, Jena, Germany) (63 × magnification, λ_Ex_/λ_Em_: 579/599 nm). A minimum of 170 mitochondria per group, obtained from randomly selected image fields, were analyzed across multiple images, and quantitative assessment of mitochondrial morphology was performed using ImageJ software. The structure of mitochondria was classified into four morphotypes based on the obtained area, perimeter, and length: long tubular (area ≥ 3 µm^2^, perimeter ≥ 2 µm, length ≥ 0.8 µm), small tubular (area ≥ 1.5 µm^2^, perimeter ≥ 1 µm, length ≥ 0.4 µm), large spherical (area ≥ 1 µm^2^, perimeter ≥ 1 µm, length ≥ 0.2 µm), and small spherical (area ≥ 0.3 µm^2^, perimeter ≥ 0.3 µm, length ≥ 0.1 µm).

### Mitochondrial respiration

To evaluate mitochondrial function and bioenergetic health in live cells, oxygen consumption rate (OCR) was measured using the Seahorse XF Cell Mito Stress test kit (Agilent Technologies, Santa Clara, CA, USA) on a Seahorse XF96 Analyzer (Agilent Technologies)^37^. Cells were cultured at a concentration of 1.5 × 10^4^ cells/well in an XF 96-well plate overnight. After treatments, the growth medium was replaced with 180 μL/well of XF base medium containing 1 mM pyruvate, 2 mM L-glutamine, and 10 mM glucose. The cells were then sequentially stimulated with oligomycin (1 μM; an ATP synthase inhibitor), FCCP (2 μM; an oxidative phosphorylation uncoupler), and rotenone/antimycin (0.5 μM; a mitochondrial complexes I and III inhibitor). At designated time points, the OCR was measured and the following parameters were calculated: OCR, basal respiration, maximal respiration, spare respiratory capacity, and ATP production.

### Glycolytic metabolism

To quantify the capacity and flexibility of glycolytic metabolism in real time, extracellular acidification rate (ECAR) was analyzed by employing the Seahorse XF Glycolysis Stress test kit (Agilent Technologies) with a Seahorse XF96 Analyzer^37^. An XF 96-well plate was seeded with cells at a concentration of 1.5 × 10^4^ cells per well and incubated overnight to allow adherence and growth. After treatments, the growth medium was replaced with 180 μL/well of XF base medium containing 2 mM L-glutamine. The cells were then sequentially induced with glucose (10 mM; a glycolytic substrate), oligomycin (1 μM), and 2-deoxy-D-glucose (2-DG) (50 mM; a glycolysis inhibitor). Glycolytic parameters, including ECAR, glycolysis, and glycolytic capacity, were determined following completion of the assay reactions.

### mRNA expressions by quantitative real-time PCR (RT-qPCR)

Following cell lysis, total RNA was isolated using the GeneJET RNA Purification Kit (Thermo Fisher Scientific)^35^. Quantification of mRNA expression was performed using one-step RT-qPCR with the KAPA SYBR FAST kit (KAPA Biosystems, Boston, MA, USA) on the CFX96 Real-Time PCR Detection System (Bio-Rad, Hercules, CA, USA). Primers were sequenced from Macrogen (Seoul, Republic of Korea) and were employed to assess the mRNA expression of genes involved in mitochondrial function (NRF1, PGC1α, ATP5A), mitochondrial dynamics (OPA1, MFN1, DNM1, FIS1), apoptosis (Bcl-2, BAX), and inflammation (TNFα, IL6) (Table 1). Expression levels of target genes were normalized to glyceraldehyde-3-phosphate dehydrogenase (GAPDH), and fold changes in mRNA expression were calculated using the 2^−ΔΔCt^ method.

**Table 1:** Lists of primer pairs used for RT-qPCR (Rat).

Gene target	Forward (5′-3′)	Reverse (5′-3′)
ATP5A	5′-CTTAACCAGGAACGGAAGCAGG-3′	5′-GTTGGAGTTGTCGTGTTTGGGA-3′
BAX	5′-CTGCAGAGGATGATTGCTGA-3′	5′-GATCAGCTCGGGCACTTTAG-3′
Bcl-2	5′-GCTACGAGTGGGATACTGG-3′	5′-GTGTGCAGATGCCGGTTCA-3′
DNM1	5′-ATCCAGCTGCCTCAGATTGT-3′	5′-GTGACCACACCAGTCCCTCT-3′
FIS1	5′-GAAGTATGTGCGGGGACTGT-3′	5′-CCATGCCTACCAGTCCATCT-3′
GAPDH	5′-GTGGACCTCATGGCCTACAT-3′	5′-TGTGAGGGAGATGCTCAGTG-3′
IL6	5′-AGGATACCACCCACAACAGACC-3′	5′-CACCCTCAACACACACACACCA-3′
MFN1	5′-GAGGGAAGACCAAATCGACA-3′	5′-CAGACAGGCGACAAATCTCA-3′
NRF1	5′-CCTGCTTTCAGTCCTTCTGG-3′	5′-ATGGACCTGCTGAACTTGCT-3′
OPA1	5′-TTGGGAGACCCTACAAGACG-3′	5′-GTCTTCTGCGAAGTCGTTCC-3′
PGC1α	5′-ATGTGTCGCCTTCTTGCTCT-3′	5′-CGAGAAAAGGATCTCGAACG-3′
TNFα	5′-TGCCTCAGCCTCTTCTCATTCC-3′	5′-CTCCCCGCTTACTTGCTTGTT-3′

### Protein expressions by western blotting

The protein was harvested from cells as described previously^38^. Protein extraction was performed using radio-immunoprecipitation assay (RIPA) buffer supplemented with protease inhibitor cocktail (Thermo Fisher Scientific). After centrifugation, the protein samples were collected and quantified using the BCA protein assay kit (Thermo Fisher Scientific). Each protein sample (20 µg) was loaded onto a 10% SDS-PAGE gel and subjected to electrophoresis. The separated proteins were transferred onto the polyvinylidene difluoride membrane (Bio-Rad). Afterward, the membranes were incubated with specific primary antibodies including TNFα (Santa Cruz Biotechnology, Santa Cruz, CA, USA; sc-52746, RRID:AB_630341), OPA1 (sc-393296, RRID:AB_3101815), DNM1 (sc-12724, RRID:AB_2230650), NRF1 (sc-515360, RRID:AB_3738540), ATP5A (sc-136178, RRID:AB_2061764), Bcl-2 (Cell Signaling Technology, Danvers, MA, USA; cst#28150, RRID:AB_3674827), Akt (cst#4691, RRID:AB_915783), p-Akt (Ser473) (cst#4060, RRID:AB_2315049), p-Erk1/2 (Thr202/Tyr204) (cst#4370, RRID:AB_2315112), Erk1/2 (cst#4695, RRID:AB_390779), and GAPDH (cst#5174, RRID:AB_10622025). The immunoblots were probed again with related horseradish peroxidase-conjugated secondary anti-rabbit (sc-2357, RRID:AB_628497) and anti-mouse (sc-516102, RRID:AB_2687626) antibodies. Protein signals were detected using the SignalFire ECL reagent (Cell Signaling Technology) and imaged using the iBright FL1500 Imaging System (Thermo Fisher Scientific). The signals were quantified using ImageJ software. Relative changes in target protein expression were calculated after normalization to GAPDH levels.

### Statistical analysis

Results are presented as means ± SD, calculated from at least four independent experiments. Normality of data distribution was assessed using the Shapiro–Wilk test and homogeneity of variance was evaluated using the Brown-Forsythe test. Data that satisfied these assumptions were analyzed using Student’s t-test or one-way ANOVA followed by Tukey’s post hoc test, as appropriate, using GraphPad Prism 8.0.2 software (San Diego, CA, USA). Statistical significance was defined as p < 0.05.

## Results

### Ambrisentan mitigates cisplatin-induced apoptosis and inflammation under basal and stress conditions

**Fig. 1:**
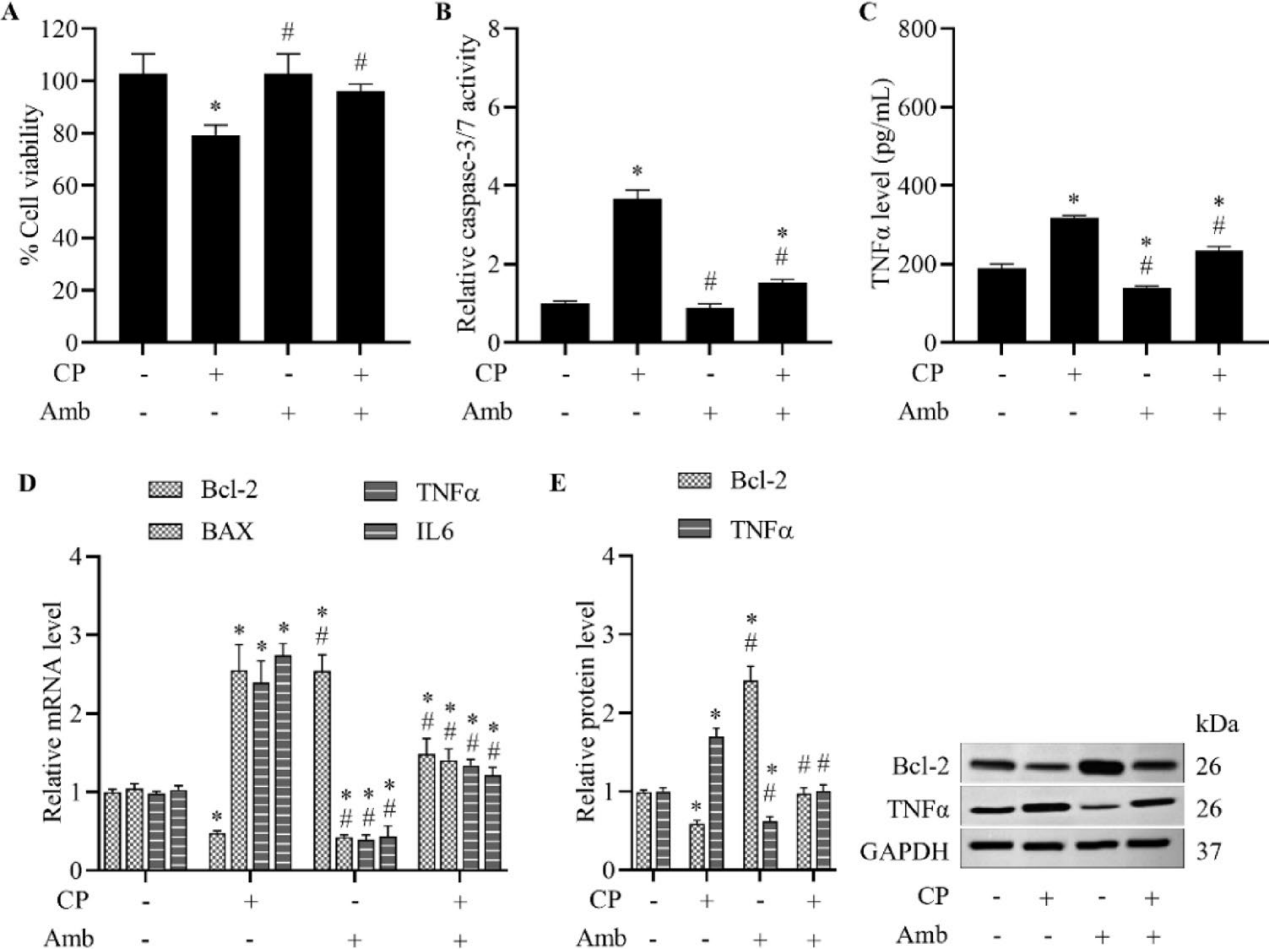
Ambrisentan diminishes cisplatin-induced apoptosis and inflammatory responses in H9c2 cells. Cells were treated with cisplatin (CP, 10 µM), ambrisentan (Amb, 1 µM), or pre-treated with ambrisentan followed by cisplatin exposure. Vehicle-treated cells served as the control group. (**A**) Cell viability was assessed using the MTT assay. (**B**) Apoptotic activity was measured using a caspase-3/7 luminescence assay. (**C**) TNFα levels were quantified using ELISA to assess the inflammatory response. (**D**) mRNA expressions of genes associated with apoptosis (Bcl-2, BAX) and inflammatory responses (TNFα, IL6) were determined by RT-qPCR. (**E**) Protein levels of Bcl-2 and TNFα were examined by western blotting. Data are expressed as mean ± SD (*N* = 4). *p < 0.05 vs control, ^#^*p* < 0.05 vs cisplatin-treated group.

To investigate the potential protective effects of ambrisentan against cisplatin-induced cardiotoxicity in H9c2 cardiomyoblasts, cell viability was assessed. Exposure to cisplatin (10 µM) resulted in significant cytotoxicity, which was notably restored by pretreatment with a non-toxic concentration of ambrisentan (1 µM) (Fig. 1A). To determine whether ambrisentan modulates apoptosis and inflammation in response to cisplatin, we assessed caspase-3/7 activity and TNFα levels. Cisplatin exposure markedly elevated both caspase-3/7 activity and TNFα levels relative to vehicle-treated controls. Under basal conditions, ambrisentan reduced the expression of these markers induced by cisplatin, suggesting its intrinsic anti-apoptotic and anti-inflammatory properties. Furthermore, ambrisentan preconditioning effectively suppressed cisplatin-induced increases in caspase-3/7 activity and TNFα expression, indicating that it counteracts the apoptotic and inflammatory responses elicited by cisplatin (Fig. 1B,C).

We further determined the expression of key mitochondrial outer membrane permeabilization (MOMP)-related genes and proteins to explore the mechanisms underlying the protective effect on apoptosis-triggering pathways. Administration with ambrisentan led to upregulation of the anti-apoptotic marker Bcl-2 and downregulation of the pro-apoptotic marker BAX, as evidenced by both mRNA and protein expression levels. In contrast, cisplatin exposure resulted in a decrease in Bcl-2 and an increase in BAX, both of which were reversed upon initial exposure to ambrisentan (Fig. 1D,E). Furthermore, cisplatin considerably increased the expression of key inflammatory markers such as TNFα and IL6, whereas treatment with ambrisentan effectively attenuated these inflammatory responses compared to vehicle-treated controls. Notably, the heightened inflammatory signals induced by cisplatin were completely abolished by prior incubation with ambrisentan (Fig. 1D,E). Overall, these results indicate that the blockade of ET_A_ receptors not only reverses the apoptotic effects of cisplatin and restores cellular survival signaling but also supports its anti-inflammatory potential in limiting cisplatin-mediated cardiac damage.

### Ambrisentan inhibits mitochondrial dysfunction and oxidative stress mediated by cisplatin

**Fig. 2:**
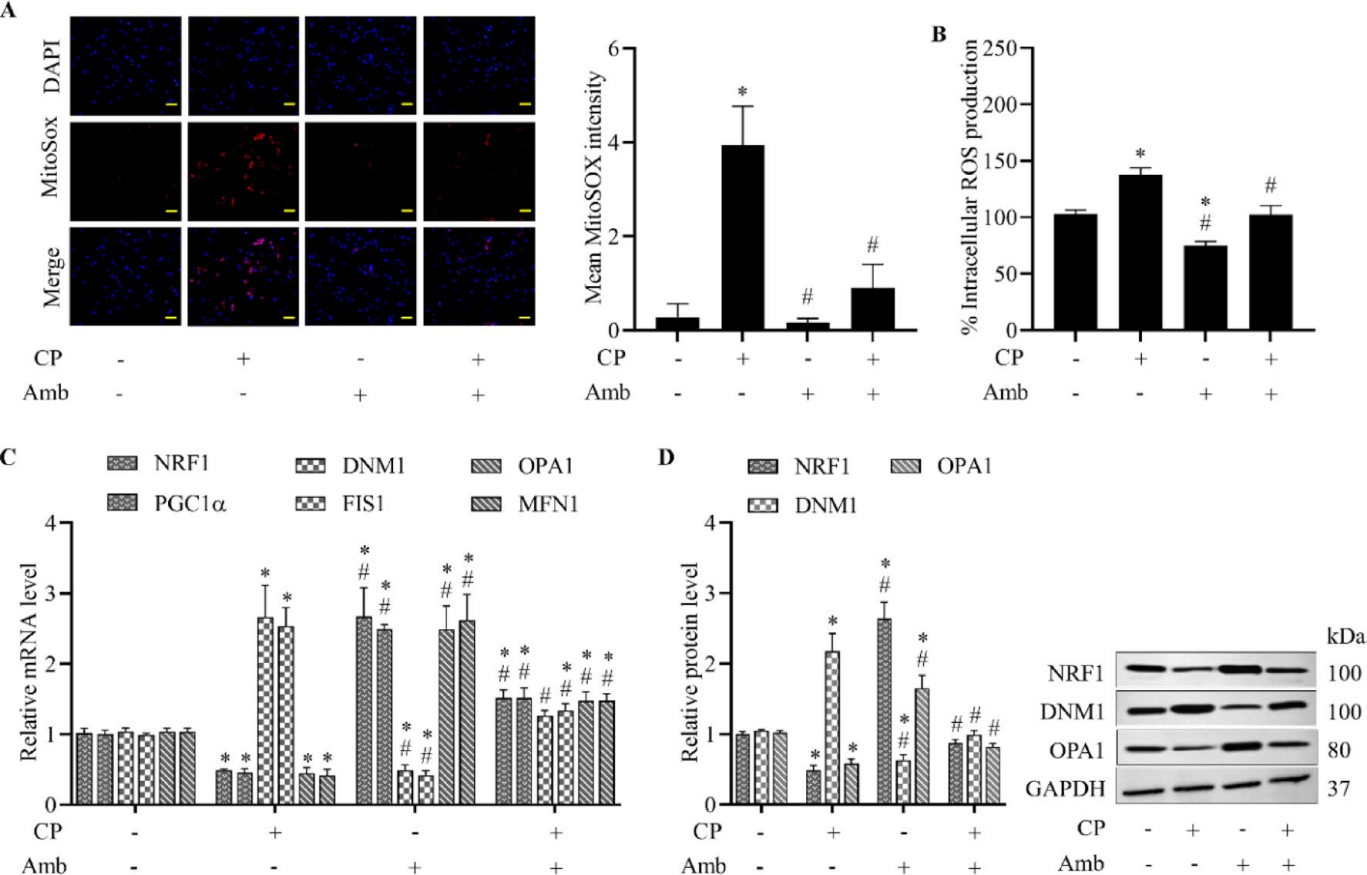
Ambrisentan suppresses mitochondrial malfunctions caused by cisplatin. Cisplatin (CP, 10 µM) and ambrisentan (Amb, 1 µM) were administered either individually or in combination, with cells receiving ambrisentan pre-treatment prior to subsequent exposure to cisplatin. Vehicle-treated cells served as controls. (**A**) Mitochondrial ROS levels were assessed using MitoSOX Red staining (20 × ; scale bar: 50 µm), with DAPI counterstaining the nuclei (blue). (**B**) Intracellular ROS levels were measured using the DCFH-DA fluorescence assay. (**C**) Relative mRNA levels of mitochondrial fission (DNM1, FIS1), fusion (OPA1, MFN1), and biogenesis (NRF1, PGC1α) markers were determined by RT-qPCR. (**D**) Protein expressions of NRF1, DNM1, and OPA1 were examined using western blotting. Data are expressed as mean ± SD (*N* = 4). *p < 0.05 vs control, ^#^*p* < 0.05 vs cisplatin-treated group.

Mitochondrial dysfunction, characterized by disrupted fission, fusion, and biogenesis, impairs oxidative stress regulation and cellular integrity in cardiomyocytes, ultimately contributing to the development of various heart diseases^4–6^. Exposure to cisplatin substantially increased ROS levels in both mitochondrial and intracellular compartments. Although ambrisentan treatment alone reduced intracellular ROS without notably affecting mitochondrial ROS compared to controls, advanced treatment with ambrisentan potently diminished ROS accumulation induced by cisplatin in both compartments (Fig. 2A,B), demonstrating that blocking the ET_A_ receptors provides an antioxidant effect, enhancing cellular resilience to oxidative stress.

To further explore the mechanistic basis of mitochondrial protection, we analyzed the expression of key regulators of mitochondrial dynamics, including fission markers (DNM1, FIS1), fusion markers (OPA1, MFN1), and biogenesis regulators (NRF1, PGC1α). Cisplatin led to an upregulation of fission markers and a concurrent downregulation of fusion and biogenesis-associated genes and proteins, indicating a shift toward mitochondrial fragmentation and dysfunction (Fig. 2C,D). In contrast, pre-incubation with ambrisentan promoted mitochondrial fusion and biogenesis while reducing fission-related gene expression. Importantly, pre-exposure to ambrisentan significantly reversed cisplatin-induced dysregulation of these mitochondrial dynamic parameters, highlighting that ET_A_ receptor antagonism supports the maintenance of mitochondrial homeostasis (Fig. 2C,D).

**Fig. 3:**
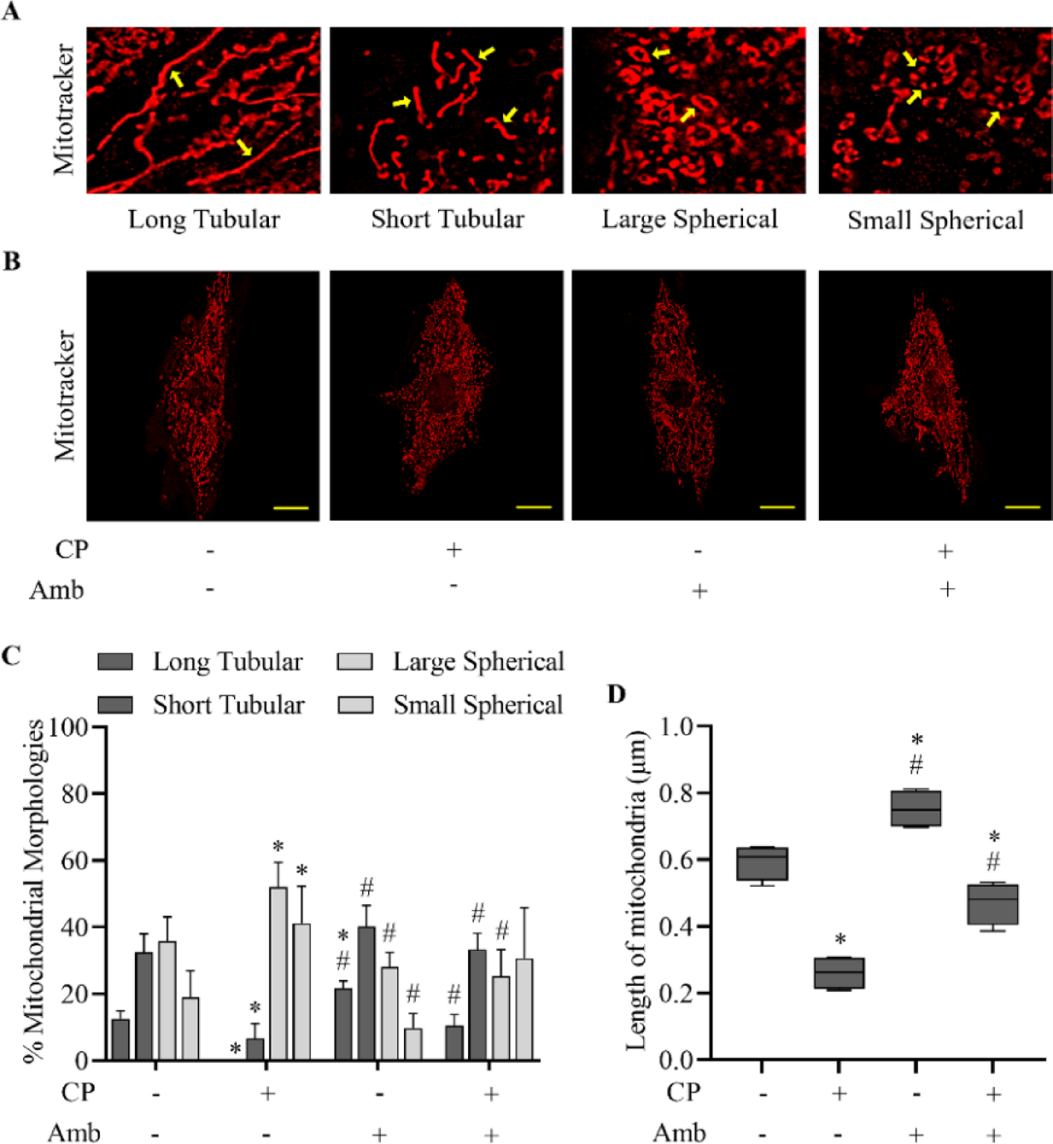
Ambrisentan preserves mitochondrial structural integrity in cisplatin-treated H9c2 cells. Treatment conditions included exposure of cells to cisplatin (CP, 10 µM), ambrisentan (Amb, 1 µM), or a sequential regimen in which cells were pre-treated with ambrisentan before cisplatin. Vehicle-exposed cells served as the control. Mitochondrial morphology was assessed by staining with MitoTracker Red CMXRos and imaging via structured illumination microscopy (SIM). (**A**) Representative images showing various mitochondrial morphologies, including long tubular, short tubular, large spherical, and small spherical shapes. (**B**) Representative images of mitochondrial morphology for each treatment group (63 × ; scale bar: 0.25 µm). (**C**, **D**) Mitochondrial morphological features, including area, perimeter, and length, were quantified using ImageJ software. Data are expressed as mean ± SD (*N* = 4). *p < 0.05 vs control, ^#^*p* < 0.05 vs cisplatin-treated group.

To assess mitochondrial structural consequences, super-resolution SIM imaging was employed to visualize mitochondrial architecture. Cisplatin-treated cells exhibited extensive mitochondrial fragmentation, with a predominance of spherical mitochondria, indicative of fission. In contrast, incubation with ambrisentan maintained an elongated and tubular mitochondrial network, consistent with enhanced fusion. Remarkably, prophylactic administration of ambrisentan mitigated the structural damage triggered by cisplatin by partially restoring mitochondrial morphology toward elongated forms (Fig. 3A–C), which was further supported by quantitative analysis of mitochondrial length (Fig. 3D). Collectively, these results successfully demonstrate the contribution of pharmacological antagonism of the ET_A_ receptors in preserving mitochondrial architecture under stress conditions.

### Ambrisentan heightens cardioprotective effects by regulation of mitochondrial and glycolytic bioenergetics disrupted by cisplatin

**Fig. 4:**
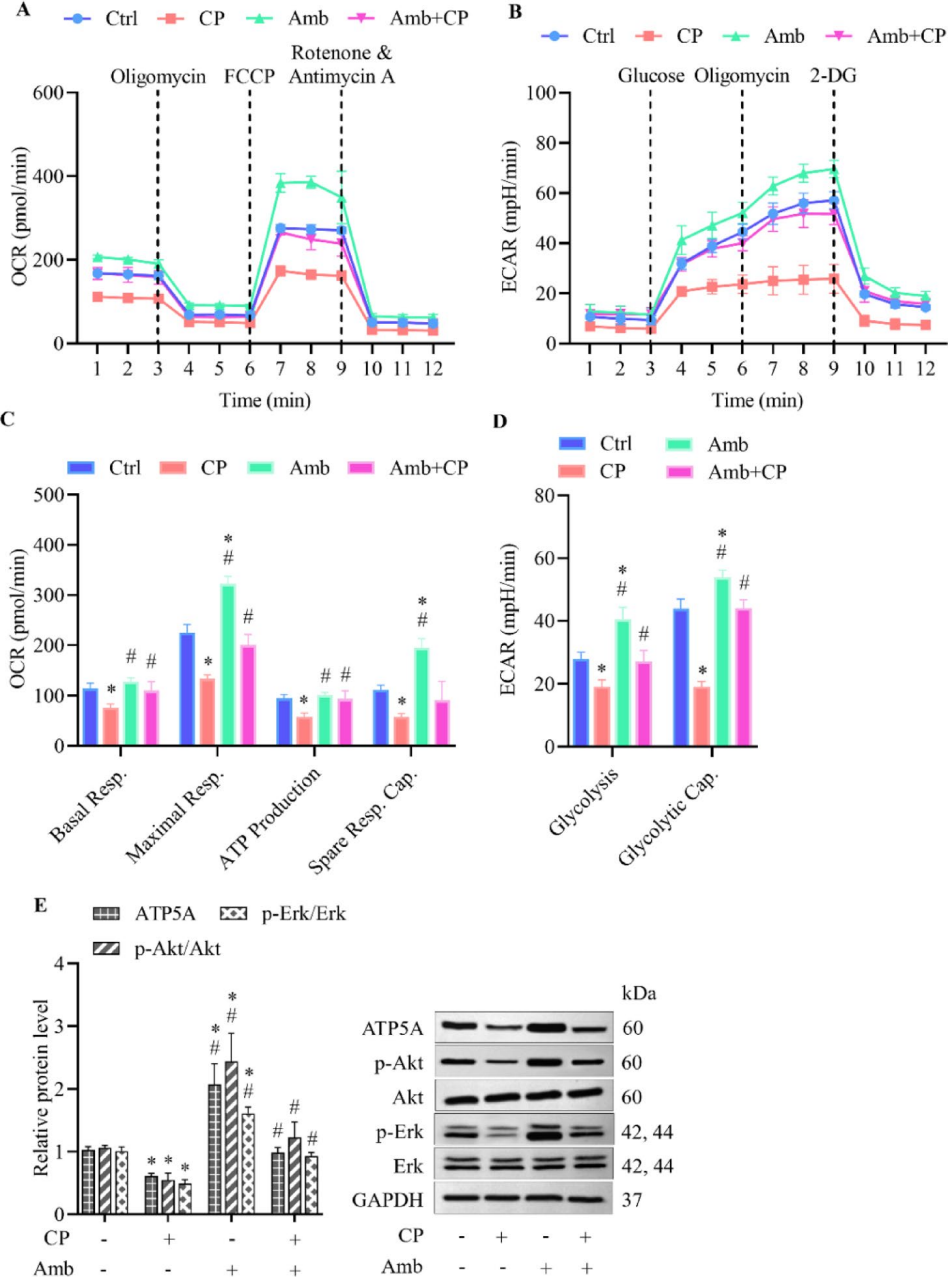
Ambrisentan boosts mitochondrial glycolytic bioenergetics in H9c2 cells exposed to cisplatin. Cells were treated with cisplatin (CP, 10 µM), ambrisentan (Amb, 1 µM), or pre-treated with ambrisentan before exposure to cisplatin. Vehicle-treated cells served as the control group. (**A**, **C**) Mitochondrial respiration was examined using the Seahorse XF Mito Stress Test to evaluate OCR parameters, including basal and maximal respiration, ATP production, and spare respiratory capacity. (**B**, **D**) Glycolytic activity was investigated using the Seahorse XF Glycolysis Stress Test to track ECAR, which reflects glycolysis and glycolytic capacity. (**E**) Protein expression levels of ATP5A and pro-survival signaling markers (p-Akt/Akt and p-Erk1/2/Erk1/2) were quantified via western blotting. Data are expressed as mean ± SD (*N* = 4). *p < 0.05 vs control, ^#^*p* < 0.05 vs cisplatin-treated group.

Glycolysis and oxidative phosphorylation are key pathways for ATP production and redox homeostasis^39,40^. Extracellular flux analysis was conducted to evaluate cellular metabolic activity, specifically measuring ECAR as an indicator of glycolysis and OCR as a marker of mitochondrial respiration. Cells treated with cisplatin exhibited a diminished response to oligomycin injections relative to vehicle-treated controls, revealing compromised ATP production and mitochondrial dysregulation (Fig. 4A,C). Likewise, upon glucose administration, these cells demonstrated reduced ECAR levels, reflecting impaired glycolytic activity (Fig. 4B,D). In contrast, treatment with ambrisentan enhanced the cellular response to both oligomycin and glucose injections, implying increased glycolysis and ATP production. Pre-administration of ambrisentan in cisplatin-treated cells resulted in no significant alterations in response to either oligomycin or glucose compared to the vehicle-treated control, underscoring the cardioprotective role of ET_A_ receptor blockade under intense situations (Fig. 4A,B). Furthermore, cisplatin decreased multiple metabolic indicators of mitochondrial function, such as basal and maximal respiration, spare respiratory capacity, ATP synthesis, as well as glycolytic activities, including glycolysis and glycolytic capacity. Nevertheless, initial exposure to ambrisentan restored all these parameters, except spare respiration capacity, to levels comparable to those of the vehicle-treated control (Fig. 4C,D).

We further examined the expression levels of proteins regulating the mitochondrial electron transport chain via western blot analysis. Cisplatin exposure markedly diminished the expression of ATP synthase (ATP5A), indicating compromised mitochondrial bioenergetics and impaired ATP generation (Fig. 4E). Importantly, treatment with ambrisentan alone elevated ATP5A expression relative to untreated cells, suggesting that blockade of ET_A_ receptors enhances mitochondrial bioenergetic capacity even under baseline conditions. In cells preconditioned with ambrisentan prior to cisplatin exposure, ATP5A expression was effectively restored to near-normal levels, emphasizing the role of ET_A_ receptor inhibition in maintaining mitochondrial integrity under stress conditions.

Activation of the Akt and Erk signaling pathways is recognized for its role in regulating mitochondrial dynamics and ATP generation, both of which are essential for maintaining cardiomyocyte function during stress^41,42^. Cisplatin administration resulted in marked suppression of phosphorylated Akt (p-Akt) and phosphorylated Erk (p-Erk1/2), highlighting disruption of these vital pro-survival cascades. Conversely, treatment with ambrisentan led to a notable upregulation of both p-Akt/Akt and p-Erk1/2/Erk1/2, suggesting intrinsic activation of cytoprotective signaling through ET_A_ receptor inhibition. Furthermore, cells receiving ambrisentan prior to cisplatin challenge displayed substantial restoration of Akt and Erk1/2 phosphorylation, closely approximating levels in the control group (Fig. 4E). Together, these findings underscore the cardioprotective capacity of ET_A_ receptor antagonism, as it restores both mitochondrial ATP synthase levels and pro-survival signaling pathways disrupted by cisplatin-induced stress.

### Cardioprotective benefits of ambrisentan are facilitated through the p53 and NF-κB pathways

**Fig. 5:**
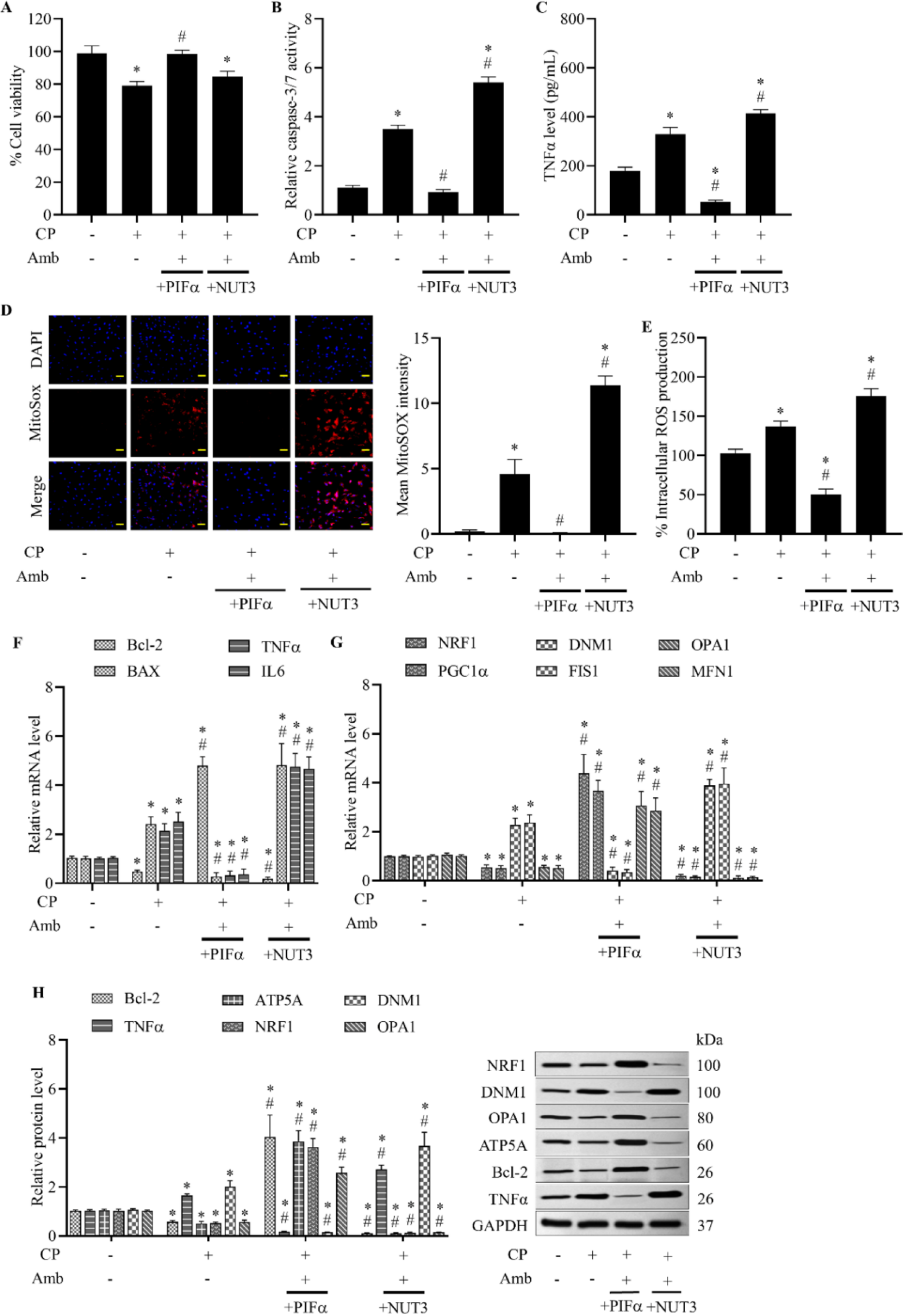
Role of the p53 in ambrisentan-mediated cardioprotection against cisplatin-induced H9c2 cell dysfunctions. Cells were pretreated with pifithrin-α (PIFα, p53 inhibitor, 20 µM) or Nutlin-3 (NUT3, p53 activator, 10 µM), followed by ambrisentan (Amb, 1 µM). Cells were then exposed to cisplatin (CP, 10 µM) for the indicated time. Vehicle-exposed cells were used as the control group. (**A**) Cell viability was assessed using the MTT assay. (**B**) Apoptotic activity was quantified via a caspase-3/7 assay. (**C**) TNFα secretion was measured using ELISA. (**D**, **E**) Mitochondrial and intracellular ROS levels were examined using MitoSOX Red (20 × ; scale bar: 50 µm) and DCFH-DA, respectively. (**F**, **G**) Relative mRNA expression of genes involved in mitochondrial biogenesis (NRF1, PGC1α), mitochondrial dynamics (OPA1, MFN1, DNM1, FIS1), apoptosis (Bcl-2, BAX), and inflammation (TNFα, IL6) was analyzed by RT-qPCR. (**H**) Western blot analyses were performed to assess protein levels of mitochondrial markers, apoptotic regulators, and inflammatory proteins. Data are expressed as mean ± SD (*N* = 4). *p < 0.05 vs control, ^#^*p* < 0.05 vs cisplatin-treated group.

**Fig. 6:**
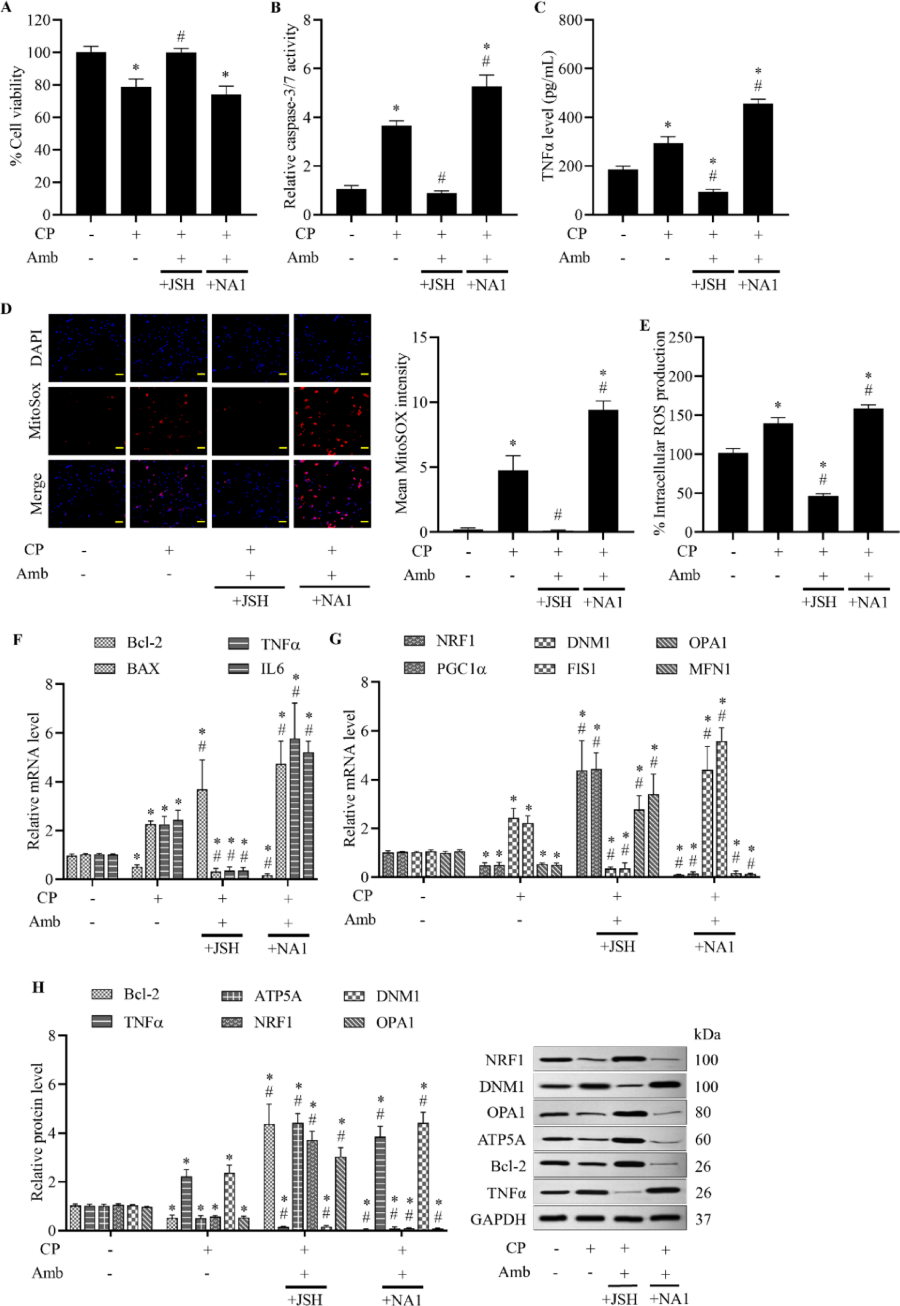
Role of the NF-κB underlying cardioprotective effects of ambrisentan in H9c2 cells stressed by cisplatin. Cells first received either JSH (NF-κB inhibitor, 10 µM) or NA1 (NF-κB activator, 5 µM), prior to incubation with ambrisentan (Amb, 1 µM); subsequently, cisplatin (CP, 10 µM) was applied. Vehicle-treated cells served as controls. (**A**) Cell viability was determined using the MTT assay. (**B**) Caspase-3/7 luminescence assay was used to measure apoptotic activity. (**C**) Inflammatory TNFα levels were quantified using ELISA. (**D**, **E**) MitoSOX Red staining (20 × ; scale bar: 50 µm) was employed to assess mitochondrial ROS levels, while intracellular ROS levels were evaluated using DCFH-DA fluorescence staining. (**F**, **G**) RT-qPCR was performed to analyze the relative mRNA expression of genes related to mitochondrial biogenesis (NRF1, PGC1α), mitochondrial dynamics (OPA1, MFN1, DNM1, FIS1), apoptosis (Bcl-2, BAX), and inflammation (TNFα, IL6). (**H**) Expression levels of proteins related to mitochondrial functions, apoptosis, and inflammatory response were assessed by western blotting. Data are presented as mean ± SD (*N* = 4). *p < 0.05 vs control, ^#^*p* < 0.05 vs cisplatin-treated group.

Ambrisentan has been shown to regulate mitochondrial biogenesis, apoptosis, and oxidative stress through the p53 and NF-κB signaling pathways^32,33^. In this study, we evaluated the role of the p53 and NF-κB pathways in mediating cardiac defensive mechanisms of ambrisentan, using specific signaling inhibitors and activators for p53 (PIFα and NUT3, respectively) and NF-κB (JSH and NA1, respectively). Co-treatment with either PIFα or JSH and ambrisentan remarkably enhanced cell survival compared to cisplatin treatment alone. Nonetheless, combined administration of either NUT3 or NA1 alongside ambrisentan led to a notable reduction in cell viability, comparable to that observed in cisplatin-treated cells (Figs. 5A and 6A), showing that the inhibition of either p53 or NF-κB pathways may abrogate the cytoprotective effects conferred by ET_A_ receptor modulation.

Apoptotic signaling, assessed via caspase-3/7 activity, was markedly suppressed by simultaneous administration of either PIFα or JSH with ambrisentan in cisplatin-challenged cells, whereas dual treatment with NUT3 or NA1 along with ambrisentan caused a significant increase in caspase-3/7 activity beyond levels observed in the cisplatin-only group (Figs. 5B and 6B). Inflammatory cytokine production, specifically TNFα levels, showed a marked decrease following co-treatment with either PIFα or JSH together with ambrisentan in cisplatin-stimulated cells. However, ambrisentan combined with either NUT3 or NA1 elevated TNFα levels relative to cells that received cisplatin (Figs. 5C and 6C). In addition, intracellular and mitochondrial ROS production was notably attenuated in cisplatin-injured cells treated with either PIFα or JSH in combination with ambrisentan. By contrast, ROS accumulation in both compartments remained high in cells co-treated with either NUT3 or NA1 and ambrisentan, even surpassing the levels observed in the group treated with cisplatin alone (Figs. 5D, E, 6D, and E). Cumulatively, these results underscore the critical involvement of both p53 and NF-κB signaling pathways in mediating the anti-apoptotic, anti-inflammatory, and antioxidative effects elicited by ET_A_ receptor antagonism.

Gene expression profiling offered molecular mechanistic insights into the cardioprotective impacts of ambrisentan. In cells challenged with cisplatin, combined treatment with either PIFα or JSH, along with ambrisentan, upregulated the expression of genes associated with anti-apoptotic (Bcl-2), mitochondrial biogenesis (NRF1, PGC1α), and mitochondrial fusion (OPA1, MFN1), while downregulating transcripts related to pro-apoptotic (BAX), pro-inflammatory (TNFα, IL6), and mitochondrial fission (DNM1, FIS1) pathways. Conversely, prior incubation with either NUT3 or NA1, along with ambrisentan, elicited an opposing gene expression profile (Figs. 5F, G, 6F, and G). Protein expression patterns mirrored the changes observed at the transcriptional level. Ambrisentan preconditioning in cisplatin-exposed cells, when combined with either PIFα or JSH, increased Bcl-2, NRF1, and OPA1 protein levels and reduced TNFα and DNM1 expression. On the contrary, ambrisentan combined with either NUT3 or NA1 in cisplatin-injured cells decreased mitochondrial and anti-apoptotic protein expression, while raising inflammatory and fission markers (Figs. 5H and 6H). These findings emphasize that the cardiac defensive properties of ET_A_ receptor blockers depend on both transcriptional and translational regulation mediated by p53 and NF-κB signaling, which are essential for maintaining mitochondrial integrity and repressing apoptotic and inflammatory gene programs.

**Fig. 7:**
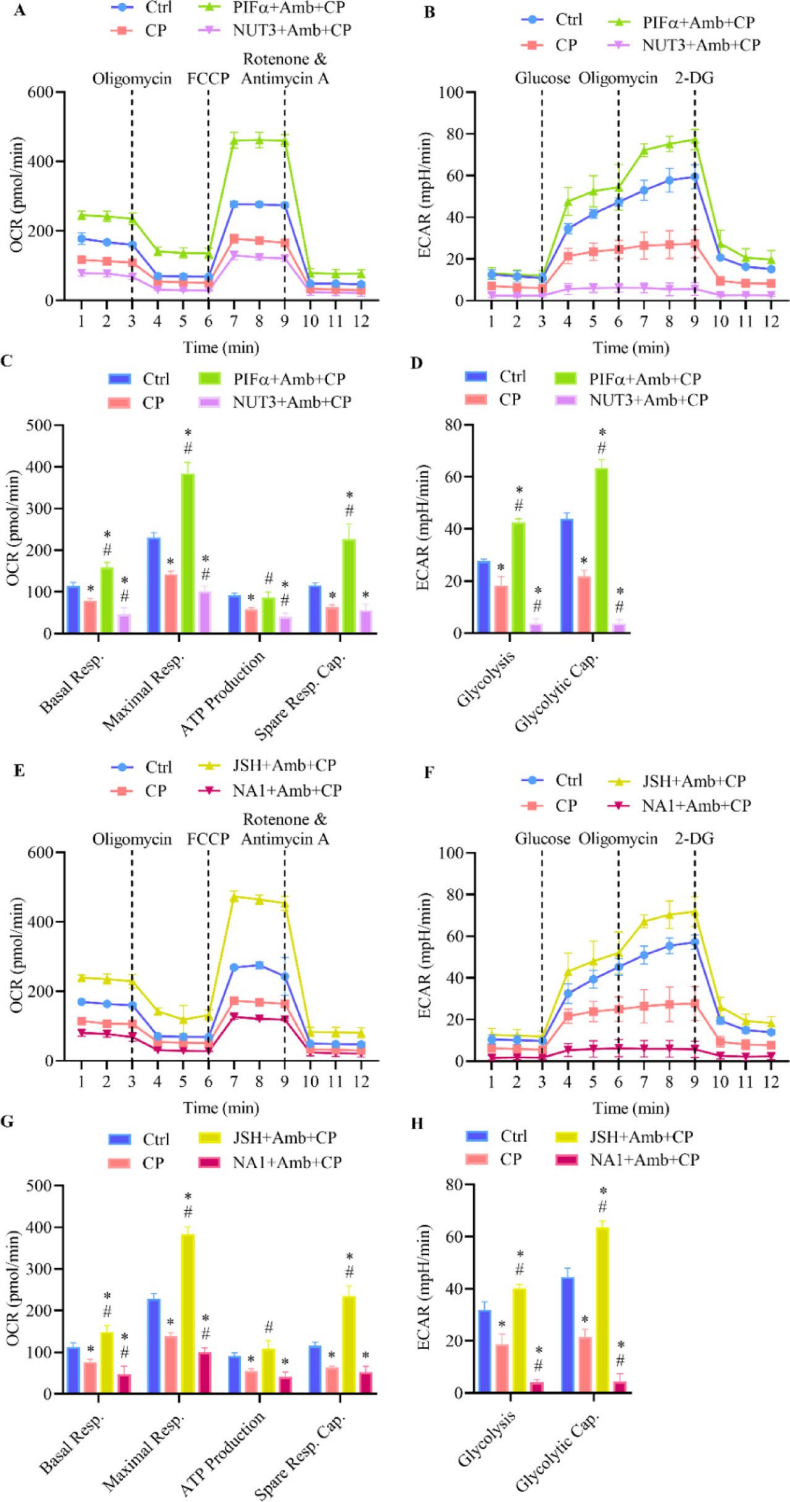
Ambrisentan regulates cellular bioenergetics via p53 and NF-κB pathways in cisplatin-treated H9c2 cells. Cells were pre-treated with ambrisentan (Amb, 1 µM) in combination with p53 (PIFα or NUT3) or NF-κB (JSH or NA1) pathway modulators before being exposed to cisplatin (CP, 10 µM). Vehicle-challenged cells served as the control group. (**A**, **C**, **E**, **G**) Mitochondrial respiration was evaluated using the Seahorse XF Mito Stress Test to measure OCR parameters, including basal and maximal respiration, ATP production, and spare respiratory capacity. (**B**, **D**, **F**, **H**) Glycolytic activity was assessed using the Seahorse XF Glycolysis Stress Test to monitor ECAR, reflecting glycolysis and glycolytic capacity. Data are expressed as mean ± SD (*N* = 4). *p < 0.05 vs control, ^#^*p* < 0.05 vs cisplatin-treated group.

Mitochondrial respiration and glycolytic function were evaluated through comprehensive bioenergetic and metabolic profiling, utilizing real-time measurements of OCR and ECAR. Cisplatin-induced impairments of mitochondrial respiration and glycolysis were reversed when ambrisentan was co-administered with either PIFα or JSH. Cells in these groups exhibited elevated OCR, ECAR, ATP production, basal and maximal respiration, spare respiratory capacity, and glycolytic capacity. On the contrary, ambrisentan treatment alongside either NUT3 or NA1 suppressed all these parameters, suggesting that disruption of either p53 or NF-κB signaling eliminates metabolic restorative effects of ambrisentan under cardiotoxic conditions (Fig. 7A–H). These outcomes substantiate the hypothesis that functional integrity of both signaling pathways is essential for enabling the metabolic restoration afforded by ET_A_ receptor inhibition.

## Discussion

Heart failure remains a multifaceted clinical syndrome marked by progressive myocardial deterioration, driven by cumulative insults such as apoptosis, inflammation, oxidative stress, and mitochondrial dysfunction^4^. Among the pivotal molecular mediators, ET-1 plays a crucial role in exacerbating cardiac injury through vasoconstriction, apoptosis, and fibrosis, primarily via activation of the ET receptors^22^. Chemotherapeutic agents like cisplatin significantly upregulate ET-1 expression, amplifying cardiotoxic pathways^43^, highlighting ET antagonism as a promising cardioprotective strategy during chemotherapy. To the best of our knowledge, the present study provides the first evidence that ambrisentan effectively mitigates cisplatin-induced cardiotoxicity in H9c2 cardiomyoblasts by attenuating apoptosis, inflammation, and mitochondrial dysfunction, while promoting mitochondrial biogenesis and survival signaling. These findings corroborate previous reports on the cardioprotective potential of ET blockade, emphasizing the therapeutic significance of targeting the ET-1 axis to improve cardiac remodeling prior to chemotherapy and prevent chemotherapy-associated cardiac injury^44,45^.

Cardiomyocyte apoptosis is a cardinal feature of cisplatin-triggered injury, primarily mediated through the intrinsic mitochondrial pathway^46^. Our results revealed the profound impact of cisplatin in disrupting the balance of pro- and anti-apoptotic (BAX/Bcl-2) mediators, accompanied by a marked elevation of caspase-3/7 activity that favors cell death. These findings reflect the classical intrinsic apoptotic pathway, wherein MOMP, driven by pro-apoptotic Bcl-2 family members such as BAX, results in cytochrome c release and subsequent caspase activation. The upregulation of BAX, coupled with the suppression of anti-apoptotic Bcl-2, is a well-documented mechanism of chemotherapy-induced cardiac cell loss^46^. Importantly, ambrisentan reversed these effects, restoring Bcl-2 expression and suppressing BAX and caspase activation, thereby limiting mitochondrial permeability and halting the downstream apoptotic cascade. This anti-apoptotic effect aligns with the understanding that ET receptor activation facilitates apoptosis, calcium channel dysregulation, and ROS generation in cardiac tissues^47,48^. Moreover, blockade of the ET receptor pathway reduced the extent of cardiomyocyte apoptosis following ischemic injury in rat models^47^. Collectively, the findings support the hypothesis that ET_A_ receptor blockade modulates upstream pro-death signaling pathways in cardiomyocytes, even under conditions of cytotoxic insult.

Inflammatory signaling is equally critical in cisplatin-induced cardiac injury, characterized by the upregulation of cytokines, including TNFα and IL6. Inflammatory mediators not only contribute to direct cardiomyocyte injury but also exacerbate oxidative stress, disturb mitochondrial function, and perpetuate apoptosis, making their inhibition critical for cardiac protection^17^. Our data demonstrated that cells challenged with cisplatin display a pronounced increase in the inflammatory cytokines TNFα and IL6, which are key mediators of cardiac dysfunction. These cytokines are typically upregulated through activation of the NF-κB and mitogen-activated protein kinase (MAPK) pathways, which are downstream effectors of stress-induced ET receptor signaling^49,50^. Interestingly, ambrisentan suppressed TNFα and IL6 release in cisplatin-exposed H9c2 cardiomyoblasts, indicating that ET_A_ receptor antagonism interrupts inflammatory transcriptional programs. The inflammatory effect is supported by previous studies where ET agonism increased cytokine production in models of cardiac hypertrophy and diabetic nephropathy^51,52^. Consequently, the selective inhibition of ET_A_ receptors by specific antagonists may confer anti-inflammatory effects, further reinforcing their potential to limit cardiac inflammation and immune activation under chemotherapeutic stress.

Mitochondrial dynamics, including the balance between fission and fusion processes, as well as mitochondrial biogenesis, are essential for cardiomyocyte adaptation and survival under stress^53^. In this study, cisplatin exposure remarkably induced the expression of mitochondrial fission genes such as DNM1 and FIS1, while suppressing fusion markers OPA1 and MFN1, indicating a shift toward mitochondrial fragmentation. Additionally, cisplatin reduced the expression of mitochondrial biogenesis regulators PGC-1α and NRF1, along with ATP5A, suggesting impaired mitochondrial renewal and energy production. These changes mirror prior findings where cisplatin promotes mitochondrial fragmentation and suppresses biogenesis to induce cardiotoxicity^54^. Notably, prophylactic administration of ambrisentan reversed these mitochondrial alterations by restoring the expression of fusion and biogenesis-related genes while downregulating fission markers. This observation addresses a critical knowledge gap, as direct evidence on the role of ET receptor antagonism in regulating mitochondrial dynamics and biogenesis during chemotherapy-induced cardiac injury is limited and suggests that suppressing ET_A_ receptor-mediated signaling may preserve mitochondrial quality control and confer cardiac defense.

Mitochondrial morphology further reflects the functional state of these organelles and is highly sensitive to cellular stress. Fragmented and spherical mitochondria are hallmark indicators of mitochondrial dysfunction and elevated oxidative stress, often preceding apoptotic events^55^. Our observations revealed that cisplatin-treated cardiomyocytes exhibited fragmented mitochondrial networks with increased ROS generation, indicating impaired mitochondrial integrity and heightened oxidative damage. Conversely, ambrisentan preserved a more filamentous and interconnected mitochondrial network while reducing cellular ROS levels, emphasizing effective protection against mitochondrial fragmentation caused by ET-1 and counteraction of oxidative stress. In line with this study, a previous study has supported that ERAscan mitigate oxidative stress by stabilizing ROS levels in cardiomyocytes^32^. However, to date, no studies have specifically investigated the effects of ERAson mitochondrial morphology. In this context, our findings suggest that maintaining mitochondrial morphology via ET_A_ receptor blockade may contribute to cardioprotection, particularly under stress conditions such as chemotherapy-induced or ischemic injury. Notably, the ET_B_ receptor did not appear to mediate the detrimental effects of ET-1 in cardiomyocytes, as supported by previous studies indicating a minimal or limited role for ET_B_ signaling in cardiac cells^56,57^.

Cellular bioenergetics, assessed through OCR and ECAR, provide a complementary measure of cardiomyocyte metabolic function and adaptability^58^. Cisplatin exposure caused a marked decline in both OCR and ECAR, reflecting a collapse in both oxidative phosphorylation and glycolytic flux consistent with known effects of anticancer agents, which disrupt mitochondrial respiration and reserve capacity^58^. Initial treatment with ambrisentan substantially improved basal and maximal respiration, ATP production, glycolytic capacity, and spare respiratory capacity, highlighting its ability to rescue mitochondrial and glycolytic metabolism under chemotoxic conditions. These findings align with prior studies demonstrating that inhibition of ET-initiated signal transduction improved mitochondrial respiration and overall metabolic function through the modulation of complex III and V in cardiac injury models^28^. By mitigating ET-1-mediated metabolic impairments, selective ET_A_ receptor blockers like ambrisentan may hold potential for enhancing myocardial bioenergetic resilience and preventing heart failure in patients undergoing chemotherapy.

The recovery of mitochondrial and cellular function was further supported by the reactivation of pro-survival signaling through activation of Akt and Erk^59,60^. Akt activation is known to inhibit pro-apoptotic proteins, such as Bcl-2-associated agonist of cell death (BAD), murine double minute 2 (MDM2), and glycogen synthase kinase-3 beta (GSK-3β), while promoting Bcl-2 expression to enhance cell survival. It also enhances PGC-1α-mediated mitochondrial biogenesis and improves glycolytic enzyme expression^59^. Stimulation of Erk contributes to mitochondrial stabilization by reducing mitochondrial fragmentation and improving mitochondrial architecture in cardiomyocytes under ischemic conditions^60^. In agreement with earlier reports^13,46^, cisplatin exposure markedly suppressed the phosphorylation of both Akt and Erk1/2, indicating disruption of critical survival pathways. ET receptor activation has been implicated in the downregulation of these kinases, leading to oxidative stress and pro-apoptotic signaling, whereas antagonism of ET_A_ and ET_B_ receptors has been shown to reverse these effects^19^. Consistently, administration with ambrisentan in the cisplatin-mediated H9c2 cardiomyoblasts reactivated p-Akt and p-Erk1/2 signaling, supporting the hypothesis that ET_A_ receptor blockade enhances cell viability, mitochondrial function, and metabolic homeostasis by restoring these protective kinase pathways in chemotherapy-induced cardiac injury.

The observation that ambrisentan alone influences mitochondrial dynamics and pro-survival signaling implies that the ET_A_ receptor may possess constitutive activity under basal conditions. Cardiac tissues continuously produce low levels of ET-1 under physiological conditions, which may sustain ongoing ET receptor signaling^61^. By selectively blocking ET_A_ receptors, ambrisentan could increase the availability of ET-1 to activate ET_B_ receptors, which are linked to cardioprotective effects such as nitric oxide production and anti-apoptotic signaling. Supporting this notion, investigations in liver sinusoidal endothelial cells have shown that ambrisentan treatment enhances endothelial nitric oxide synthase activity and Akt phosphorylation, likely by redirecting ET-1 binding towards ET_B_ receptors^62,63^. Further studies are warranted to clarify whether the cardioprotective actions of ambrisentan are mediated through receptor crosstalk or by a redistribution of ET-1 that preferentially enhances ET_B_ receptor-driven signaling in cardiomyocytes.

The downstream signaling effects of Akt and Erk activation intersect with key regulators of cellular stress and survival, particularly p53 and NF-κB, both of which play dual roles in determining cardiomyocyte fate^64,65^. p53 is a key pro-apoptotic transcription factor, typically activated by oxidative insults^64^. In this study, cisplatin treatment induced classical features of p53-driven cardiotoxicity characterized by heightened apoptosis, elevated pro-inflammatory cytokines, increased oxidative damage, and disrupted mitochondrial function. These deleterious effects were markedly reversed by prior incubation with ambrisentan and further enhanced upon co-administration with p53 inhibitor (PIFα), leading to the restoration of OCR and ECAR levels, upregulation of mitochondrial biogenesis and fusion-related markers, as well as anti-apoptotic markers Bcl-2, alongside suppression of mitochondrial fission genes, the pro-apoptotic marker BAX, and inflammatory mediators (TNFα, IL6). These findings align with previous studies linking ET_A_ receptor antagonists to p53 inhibition, which leads to mitochondrial injury and apoptosis^33^. Moreover, the obtained results highlight the cardioprotective effects of ambrisentan via p53 signaling and ET_A_ receptor antagonism as a viable strategy to suppress p53-driven apoptosis and preserve cardiomyocyte function under chemotherapeutic stress.

Simultaneously, NF-κB signaling emerged as another pivotal mediator in cardiac defensive mechanisms, functioning alongside p53 to regulate cell death, inflammation, oxidative burden, and mitochondrial health^66^. Ambrisentan, either alone or in combination with the NF-κB inhibitor JSH, effectively dampened cisplatin-induced apoptosis, inflammation, and oxidative stress, improved mitochondrial gene expression and function, and restored cellular metabolism. However, activation of NF-κB with NA1 reversed these benefits, intensifying inflammatory damage, apoptosis, and metabolic failure of H9c2 cardiomyoblasts. These observations emphasize the harmful consequences of sustained NF-κB activation in cisplatin-triggered cardiac injury and support the concept that ambrisentan exerts its therapeutic effect by suppressing the NF-κB signaling pathway. Additionally, previous studies have shown that blocking ET_A_ receptors suppresses NF-κB activity, which in turn preserves mitochondrial efficiency and fosters cardiomyocyte viability during cellular stress^19^. Our data further suggested a reciprocal regulatory relationship between p53 and NF-κB, wherein p53-driven mitochondrial stress appears to act upstream of NF-κB activation, while persistent NF-κB signaling amplifies p53-mediated apoptosis. These findings are in accordance with prior research on ET-1 activation in the regulation of both p53 and NF-κB pathways, compounding cellular injury^19,33^. By simultaneously inhibiting both p53 and NF-κB cascades, ambrisentan disrupts this harmful interplay, positioning ET_A_ receptor antagonism as a compelling strategy for protecting the heart against chemotherapy-associated toxicity.

## Conclusion

**Fig. 8:**
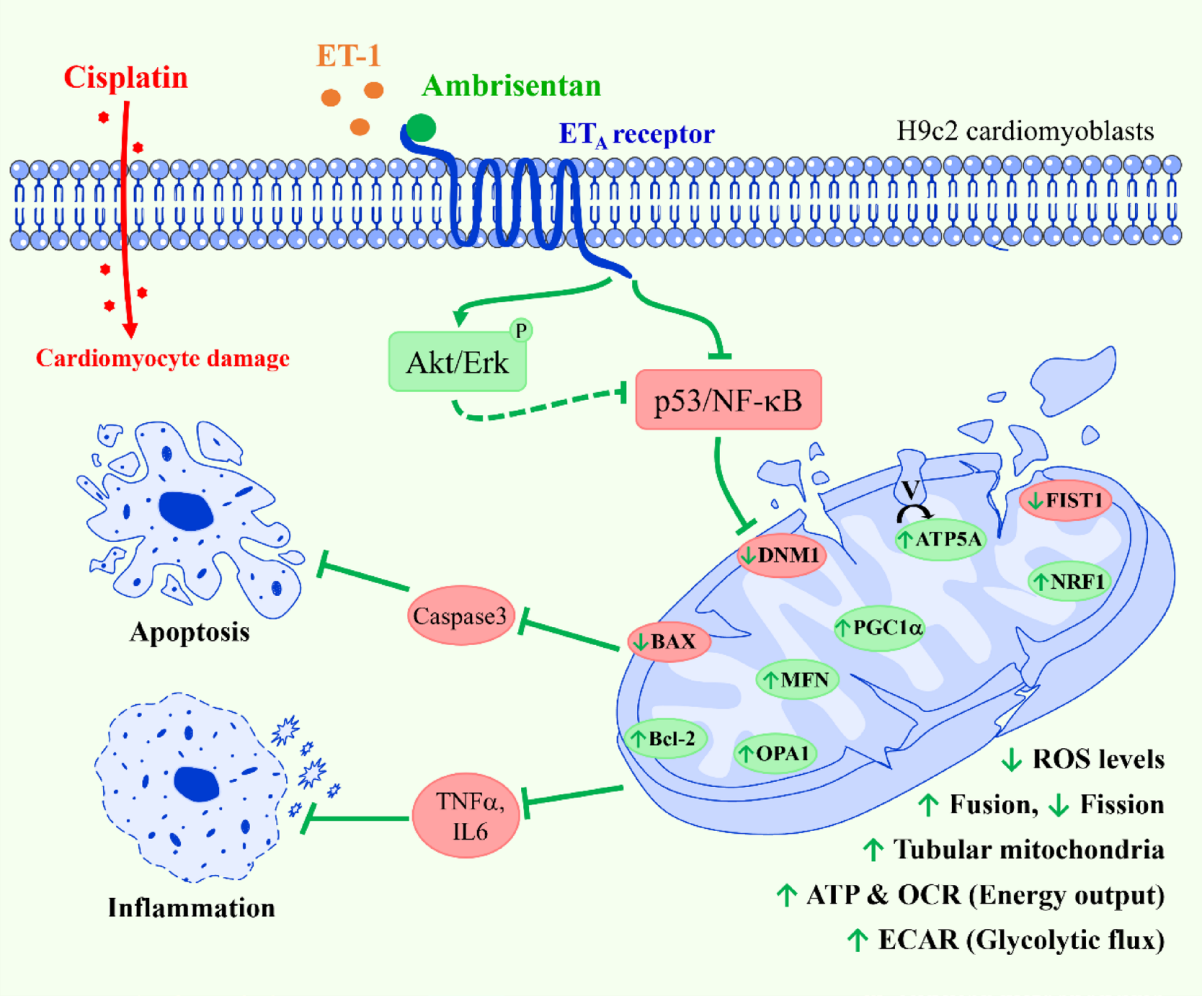
Mechanistic Overview of Ambrisentan-Mediated Cardioprotection Against Cisplatin-Induced Toxicity in H9c2 Cardiomyoblasts. In cisplatin-induced cardiotoxicity, pre-treatment with ambrisentan inhibits the binding of ET-1 to the ET_A_ receptor, thereby activating downstream pro-survival Akt and Erk signaling. Consequently, this cascade attenuates the activity of p53 and NF-κB, thereby ameliorating mitochondrial dysfunction through the promotion of mitochondrial fusion (OPA1, MFN1), stimulation of biogenesis (NRF1, PGC1α), and enhancement of ATP synthesis (ATP5A), while simultaneously suppressing mitochondrial fission (DNM1, FIS1) and reducing ROS generation. Restoration of mitochondrial homeostasis is further supported by increased mitochondrial tubularity, as well as improved OCR and ECAR. In addition, ambrisentan upregulates anti-apoptotic marker Bcl-2 as well as downregulates pro-apoptotic (BAX, caspase-3/7) and inflammatory markers (TNFα, IL-6), thereby preserving cell viability compromised by cisplatin. Solid lines denote confirmed interactions, while dashed lines indicate hypothetical interactions. Red arrows illustrate cisplatin-induced cardiotoxic pathways, whereas green arrows depict ambrisentan-mediated cardioprotective signaling.

In summary, our findings demonstrate that selective blockade of the ET_A_ receptors, particularly through ambrisentan pre-administration, represents a promising therapeutic strategy for attenuating chemotherapy-associated cardiac dysfunction, as commonly observed with cisplatin treatment (Fig. 8). Mechanistically, ambrisentan suppresses ET_A_ receptor signaling and activates the pro-survival Akt and Erk cascades, thereby attenuating apoptotic and inflammatory responses mediated by p53 and NF-κB pathways. This signaling modulation preserves mitochondrial function by restoring mitochondrial dynamics, as evidenced by the regulation of fusion (OPA1, MFN1) and fission (DNM1, FIS1) proteins, promoting biogenesis (NRF1, PGC1α, and ATP5A), and enhancing cellular energy metabolism (OCR, ECAR), ultimately resulting in elevated ATP synthesis and reduced ROS accumulation. These mitochondrial improvements are accompanied by a favorable modulation of apoptotic and inflammatory markers, including upregulation of Bcl-2 and downregulation of BAX, caspase-3/7, TNFα, and IL6, collectively supporting cardiomyocyte survival under chemotherapeutic stress. Overall, these results underscore the cardioprotective potential of ambrisentan through targeted modulation of the p53 and NF-κB signaling pathways, supporting further investigation of ET_A_ receptor antagonists as adjunctive therapies to prevent chemotherapy-induced cardiac injury. Further validation in additional cardiac cell models, such as primary cardiomyocytes or human iPSC-derived cardiomyocytes, together with subsequent in vivo studies, will be essential to strengthen the translational relevance and clinical applicability of the present findings.

## Supplementary Information

Below is the link to the electronic supplementary material.

Supplementary Material 1

## Data Availability

The data that support the findings of this study are available from the corresponding author upon reasonable request.

## Abbreviations

Akt: Protein kinase B
ATP5A: ATP synthase F1 subunit alpha
BAX: Bcl-2-associated X protein
Bcl-2: B-cell lymphoma 2
ET: Endothelin
ET-1: Endothelin-1
ERA: Endothelin receptor antagonist
DNM1: Dynamin-related protein 1
ECAR: Extracellular acidification rate
Erk: Extracellular signal-regulated kinase
FIS1: Fission protein 1
IL6: Interleukin-6
MFN: Mitofusin
NF-κB: Nuclear factor kappa B
NRF1: Nuclear respiratory factor 1
OCR: Oxygen consumption rate
OPA1: Optic atrophy 1
PGC1α: Peroxisome proliferator-activated receptor gamma coactivator-1 alpha
ROS: Reactive oxygen species
SIM: Structured illumination microscopy
TNFα: Tumor necrosis factor-alpha
