# Supplementary material for: Ambrisentan attenuates cisplatin-related mitochondrial dysfunction in the heart via regulation of p53 and NF-κB signaling
Source: Sci Rep. 2026 Mar 24;16:14850. doi: 10.1038/s41598-026-44822-9 (PMC13168273; doi:10.1038/s41598-026-44822-9)

**SUPPORTING INFORMATION**

**Supplementary Fig. S1.** Raw Western blot images corresponding to the data shown in **Figure 1**, depicting the protective effects of ambrisentan on apoptosis and inflammation in cisplatin-treated H9c2 cardiomyoblasts.


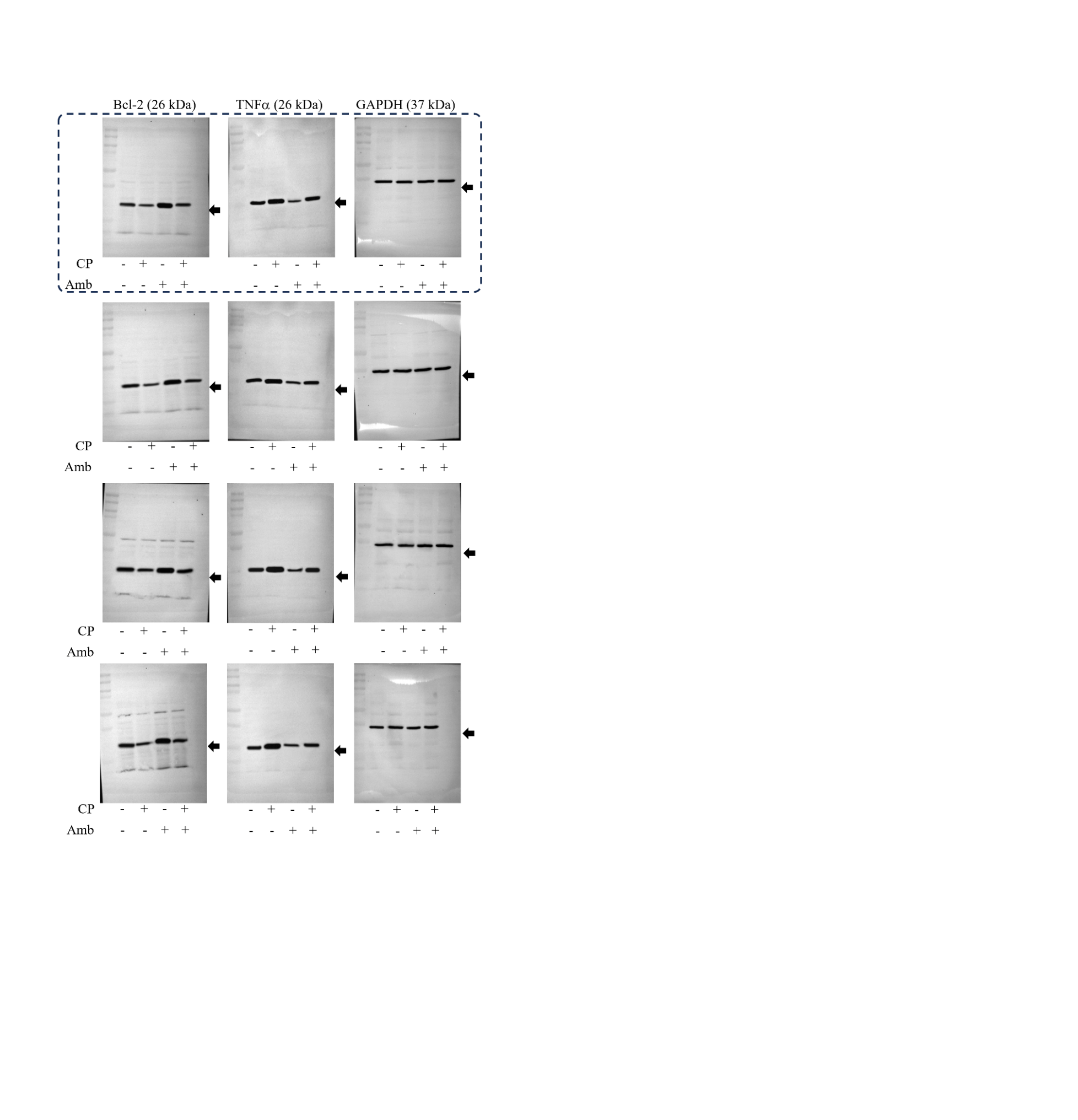


**Supplementary Fig. S2.** The Western blot images provided serve as the original source data for **Figure 2** and highlight the role of ambrisentan in preserving mitochondrial biogenesis and dynamics in H9c2 cells challenged with cisplatin.


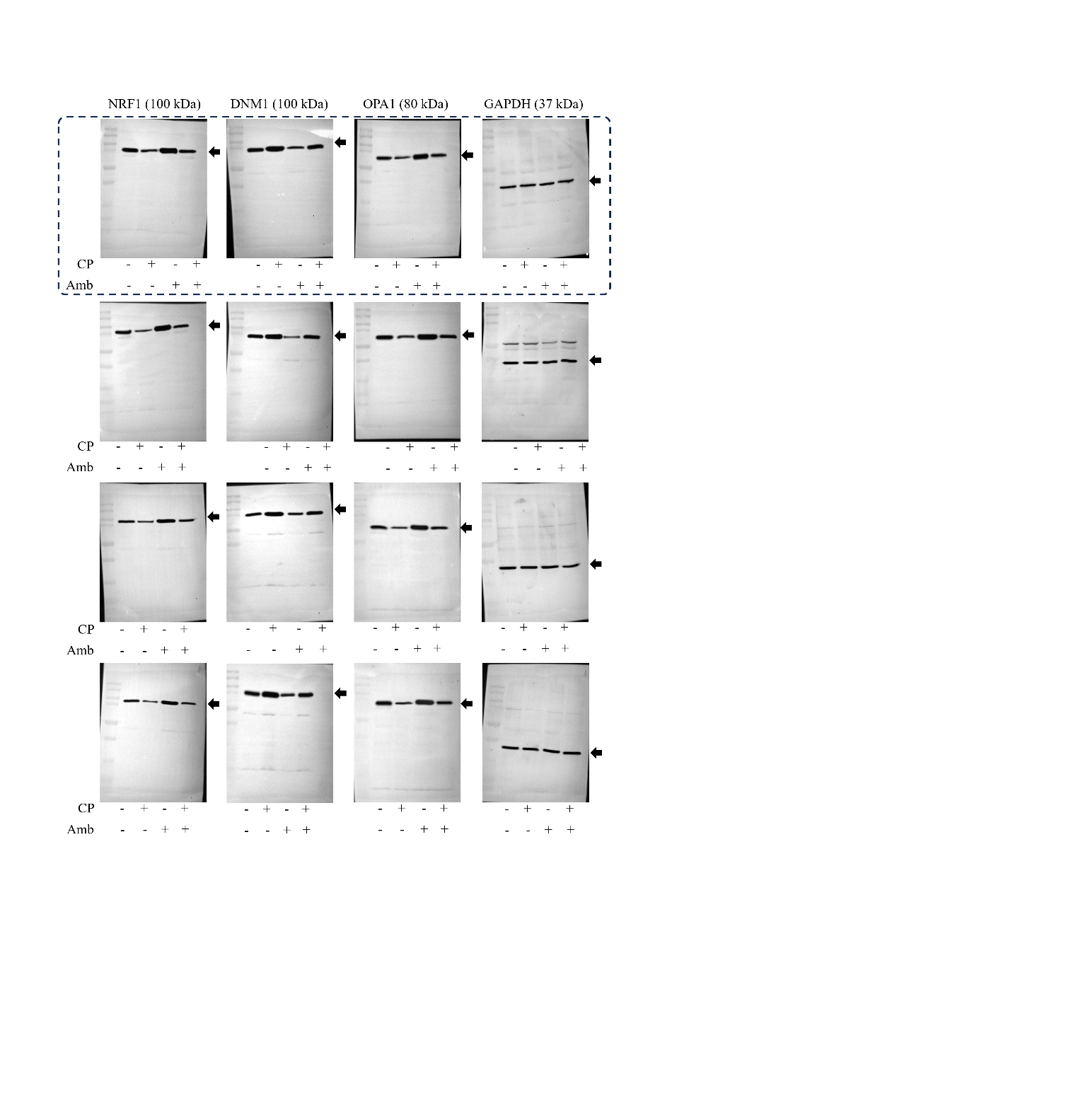


**Supplementary Fig. S3.** Uncropped Western blot images corresponding to the data presented in **Figure 4**, illustrating the role of ambrisentan in modulating pro-survival signaling and mitochondrial energy metabolism in H9c2 cells exposed to cisplatin.


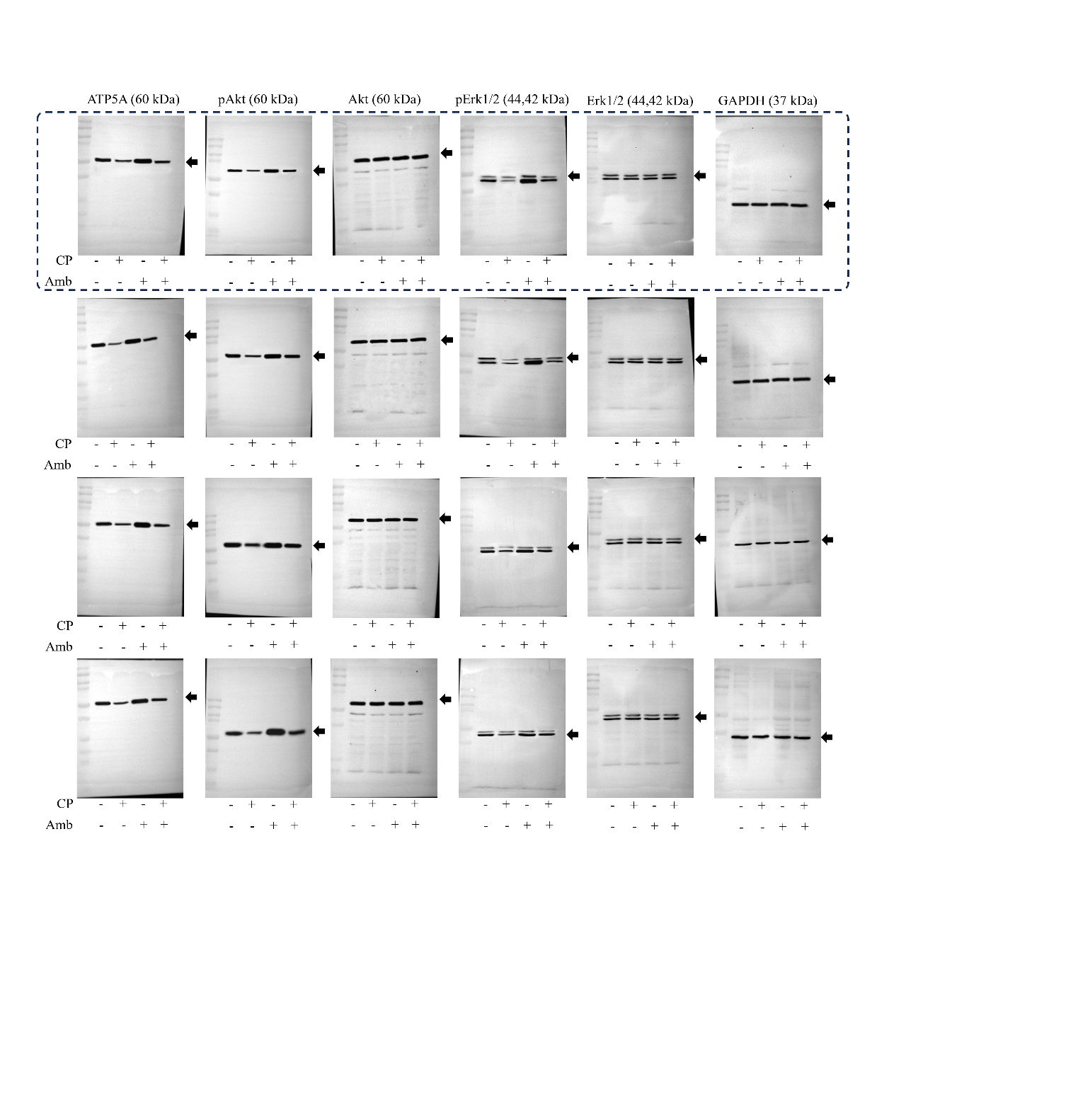


**Supplementary Fig. S4.** Unprocessed Western blot images corresponding to **Figure 5**, showing ambrisentan-mediated protection against cisplatin-induced cardiotoxicity through inhibition of the p53 signaling pathway in H9c2 cells.


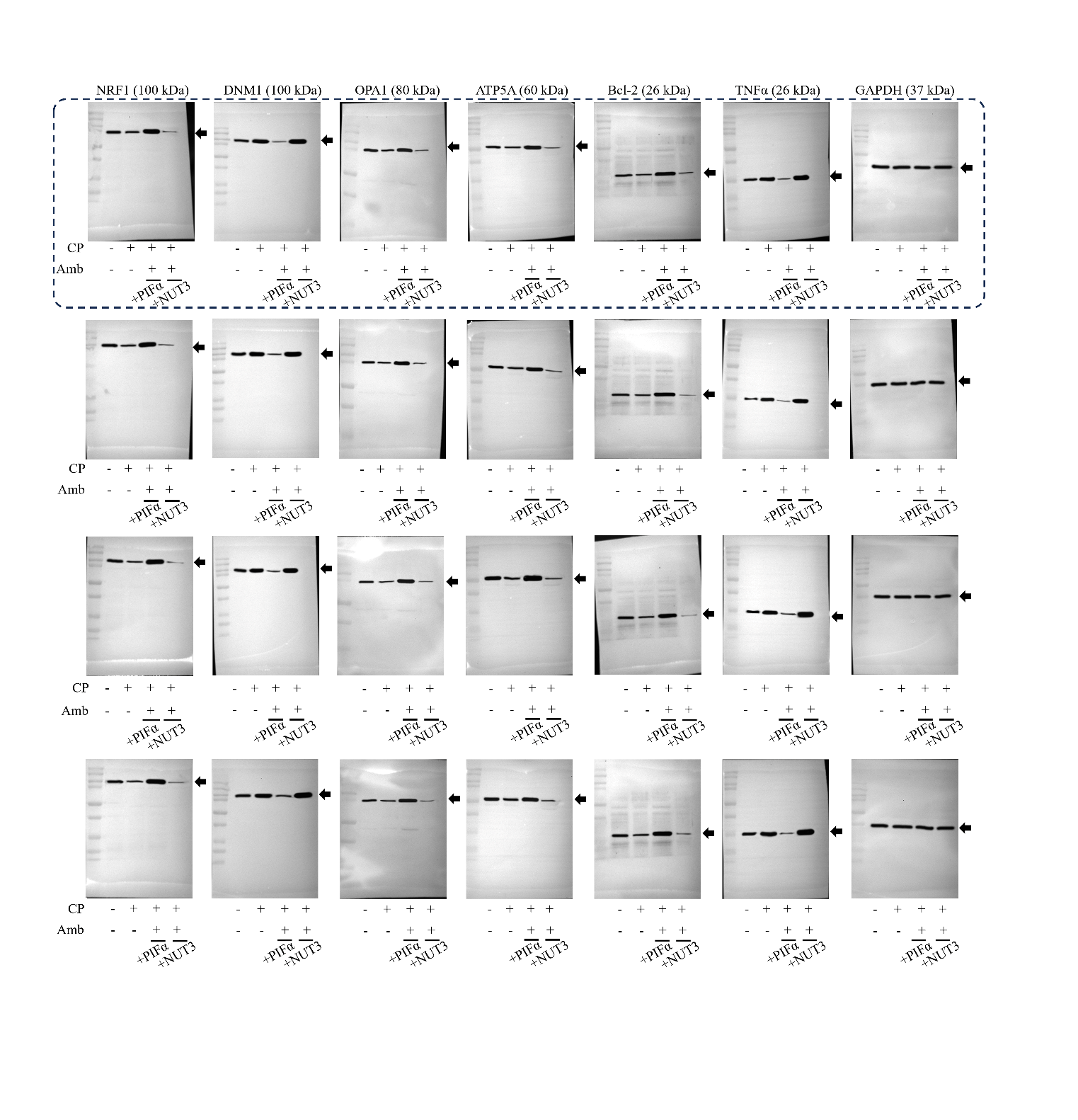


**Supplementary Fig. S5.** The unprocessed Western blot images associated with **Figure 6** demonstrate the protective effects of ambrisentan in cisplatin-treated H9c2 cells, primarily through the inhibition of the NF-κB signaling pathway


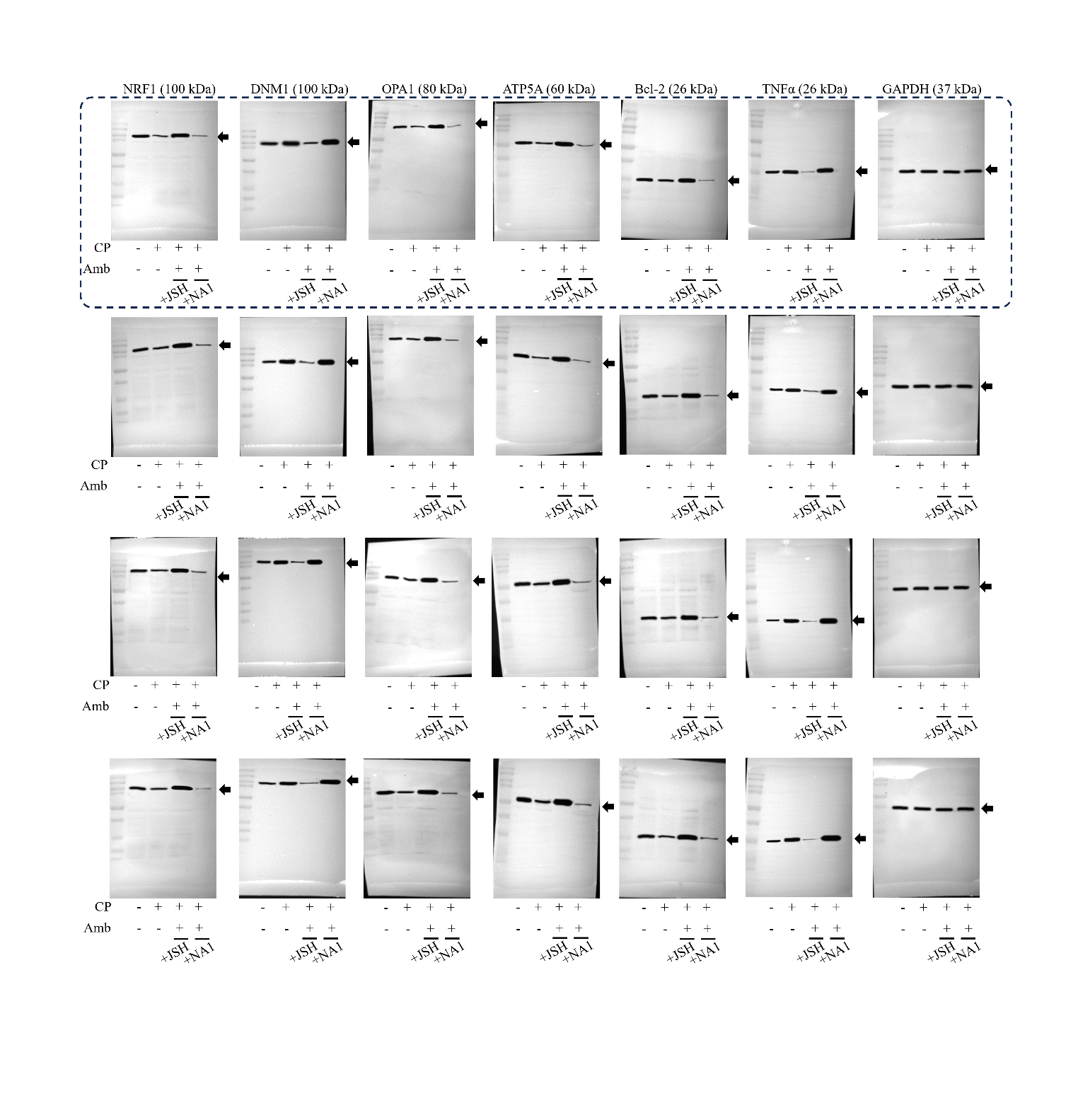

Supplement: Supplementary file 1 — Supplementary Material 1 [file 41598_2026_44822_MOESM1_ESM.doc]
